# Supplementary material for: Degradation of complex arabinoxylans by human colonic Bacteroidetes
Source: Nat Commun. 2021 Jan 19;12:459. doi: 10.1038/s41467-020-20737-5 (PMC7815789; doi:10.1038/s41467-020-20737-5)
Supplement: Supplementary file 1 — Supplementary Information [file 41467_2020_20737_MOESM1_ESM.pdf]

Supplementary Information

Degradation of complex arabinoxylans by human colonic Bacteroidetes

Pereira *et al.*

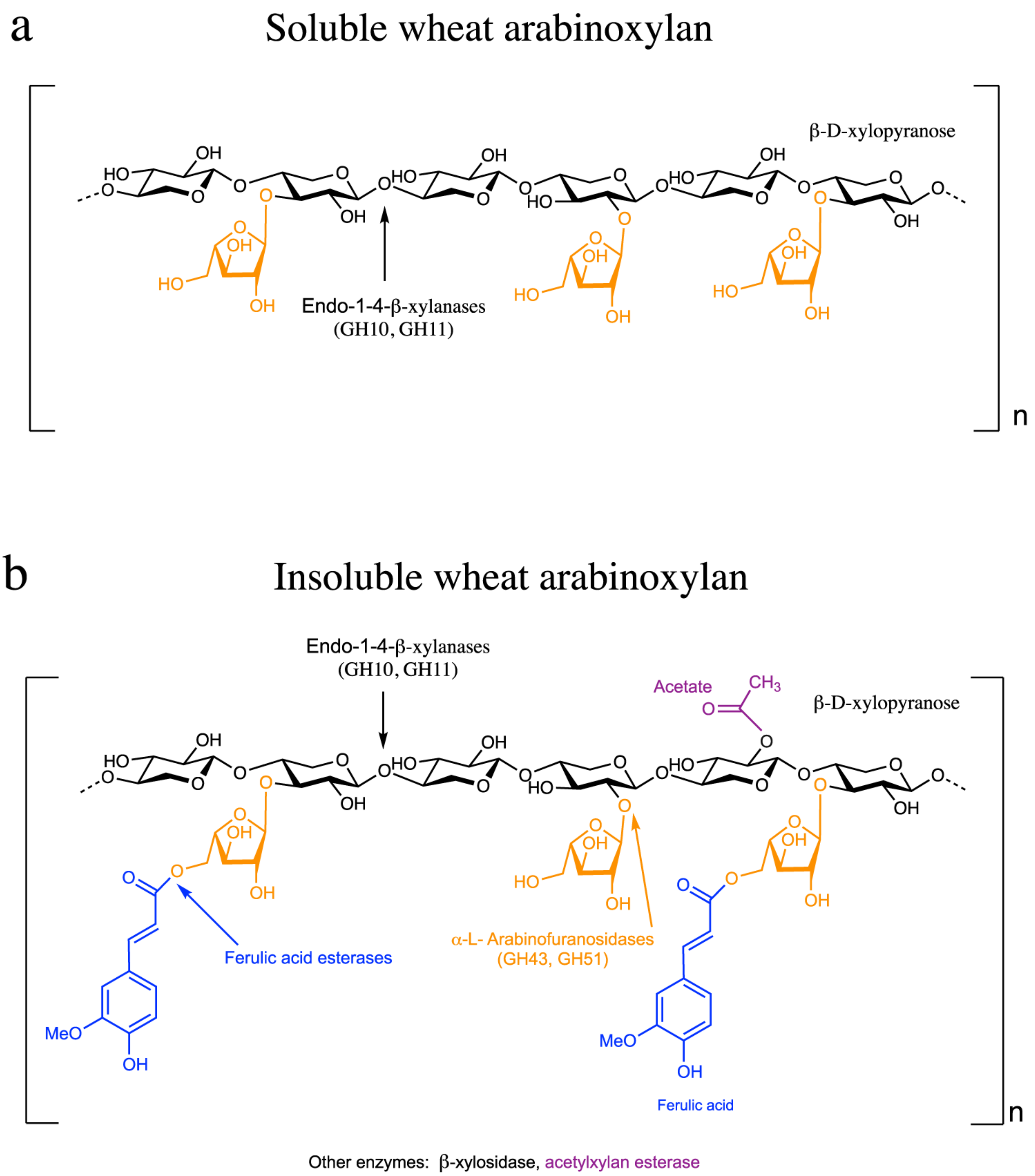
a
Soluble wheat arabinoxylan
β-D-xylopyranose
Endo-1-4-β-xylanases
(GH10, GH11)
n
b
Insoluble wheat arabinoxylan
Endo-1-4-β-xylanases
(GH10, GH11)
Acetate
β-D-xylopyranose
Ferulic acid esterases
α-L- Arabinofuranosidases
(GH43, GH51)
Ferulic acid
n
Other enzymes: β-xylosidase, acetylxylan esterase

Supplementary Fig. 1. Schematic showing the differences in soluble and insoluble wheat arabinoxylan. a Schematic structure of the soluble arabinoxylan used in this study showing the β-1,4-linked xylose backbone with arabinose side chains, but absence of ferulic acid. **b** Schematic structure of the insoluble arabinoxylan used in this study showing a similar structure to soluble arabinoxylan but with ferulic acid side chains. Common enzymes known to cleave some of the linkages in arabinoxylan are indicated, including GH10, GH11 families of endoxylanases, GH43, GH51 α-arabinofuranosidases, Ferulic acid esterase, acetyl xylan esterase (catalyzes cleavage of backbone acetyl substituents) and β-xylosidase (cleaves xylo-oligosaccharides into xylose). The n indicates the polysaccharide length is long and variable.

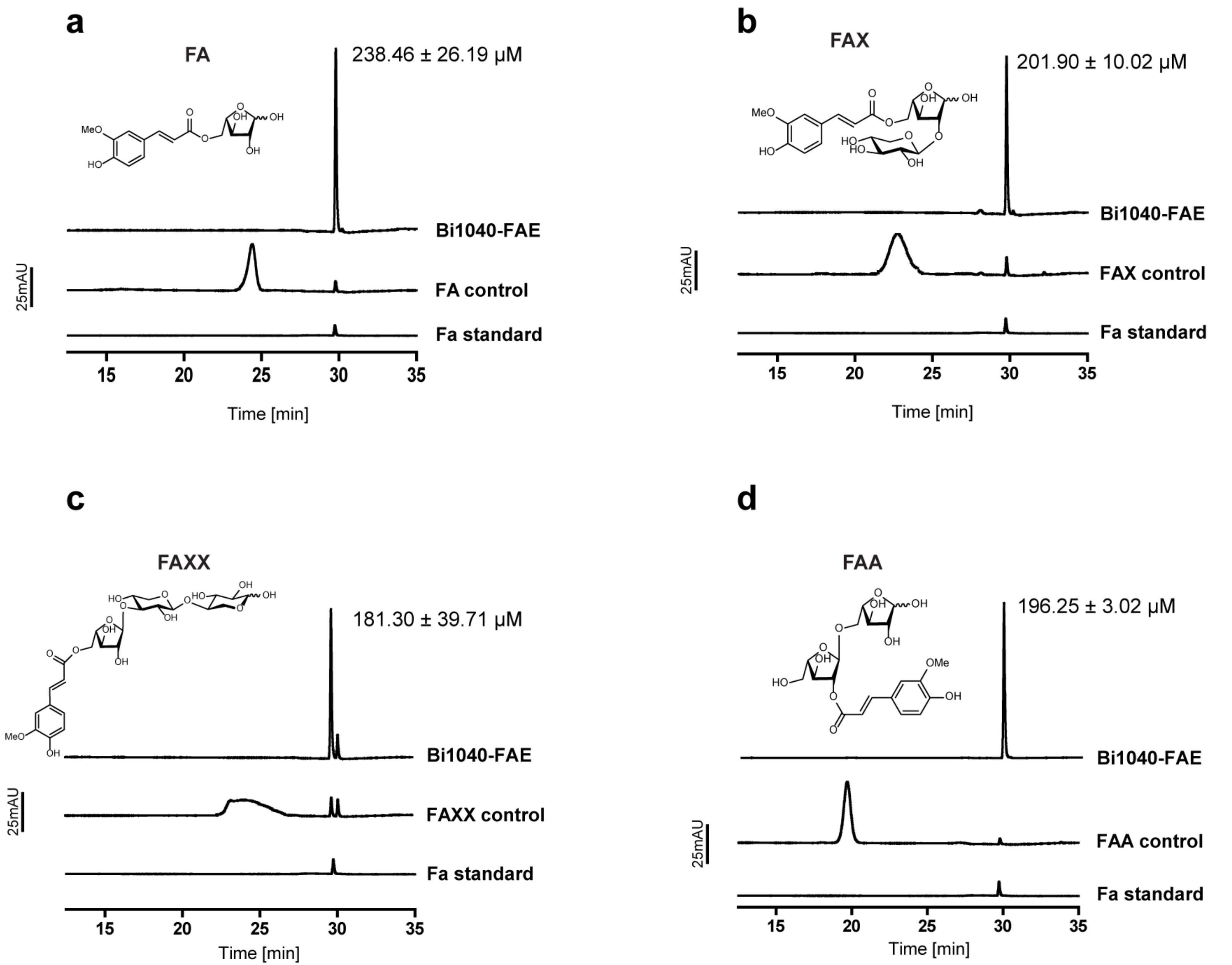
a
FA
238.46 ± 26.19 μM
Bi1040-FAE
FA control
Fa standard
25mAU
15
20
25
30
35
Time [min]
b
FAX
201.90 ± 10.02 μM
Bi1040-FAE
FAX control
Fa standard
25mAU
Time [min]
c
FAXX
181.30 ± 39.71 μM
Bi1040-FAE
FAXX control
Fa standard
25mAU
Time [min]
d
FAA
196.25 ± 3.02 μM
Bi1040-FAE
FAA control
Fa standard
25mAU
Time [min]

Supplementary Fig. 2. Cleavage of ferulic acid from feruloylated oligosaccharides by Bi1040-FAE. The hydrolytic reactions contained 50 nM of enzyme (Bi1040-FAE) with 200 μM of the respective feruloylated oligosaccharide (with structure shown). **a** Incubated with FA (ferulic acid linked to arabinose). **b** Incubated with FAX (ferulic acid linked to a disaccharide of arabinose and xylose). **c** Incubated with FAXX (ferulic acid linked to a trisaccharide of arabinose and two xylose residues). **d** Incubated with FAA (ferulic acid linked to arabinobiose). All reactions were carried out for 10 minutes at 37 °C in a buffer of pH 6.5. The end products were analyzed by C18-HPLC, as described in the Methods section. Fa: ferulic acid. The y-axis is absorbance in milli-absorbance units (mAU). The results are presented as the mean ± the standard deviation of three independent reactions (n=3), and the traces are representatives of the three different reactions. The source data underlying Supplementary Fig. 2 are provided in the Source Data file.

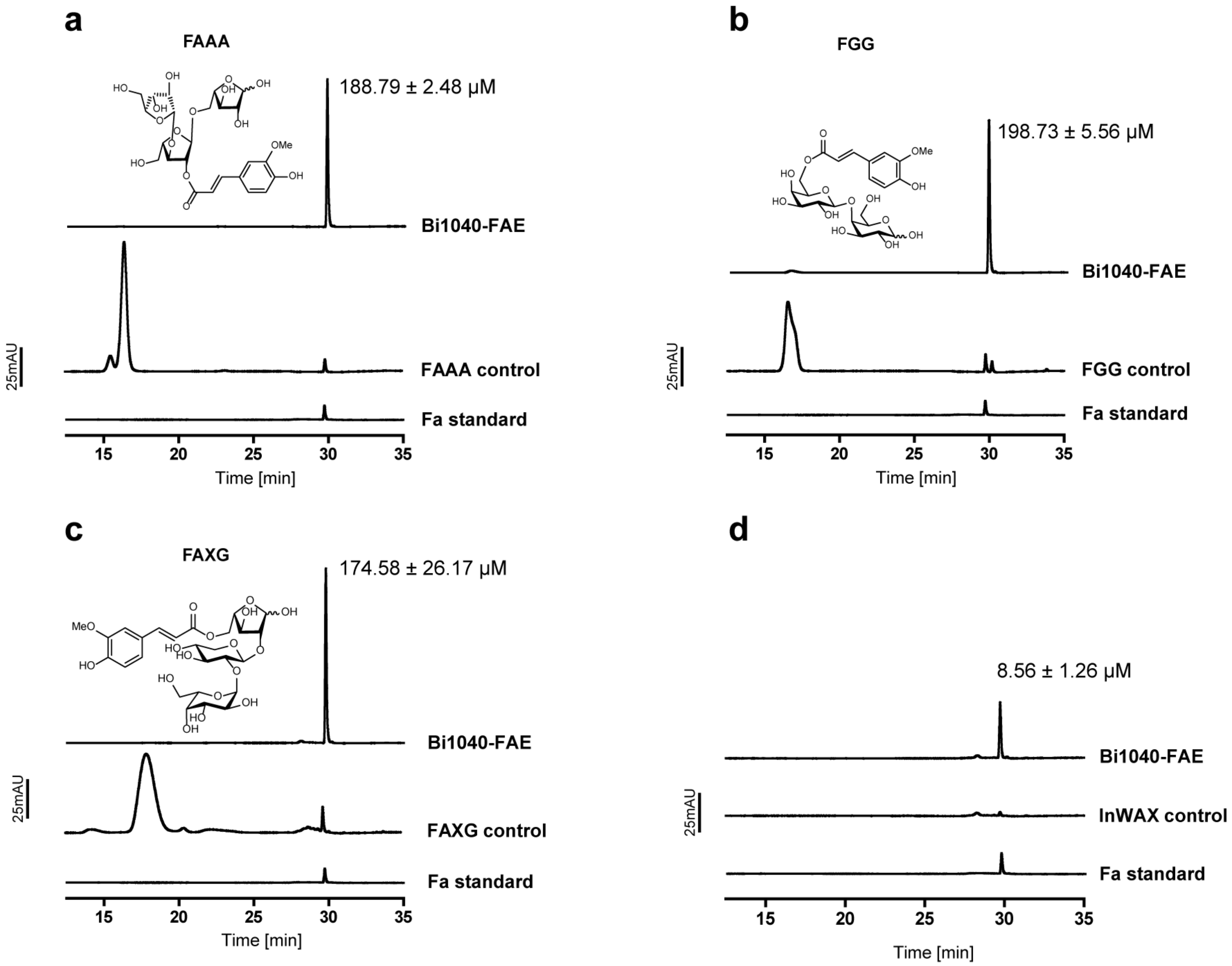

a
FAAA
188.79 ± 2.48 µM
Bi1040-FAE
FAAA control
Fa standard
25mAU
Time [min]
b
FGG
198.73 ± 5.56 µM
Bi1040-FAE
FGG control
Fa standard
25mAU
Time [min]
c
FAXG
174.58 ± 26.17 µM
Bi1040-FAE
FAXG control
Fa standard
25mAU
Time [min]
d
8.56 ± 1.26 µM
Bi1040-FAE
InWAX control
Fa standard
25mAU
Time [min]

Supplementary Fig 3. Cleavage of ferulic acid from feruloylated oligosaccharides and InWAX by Bi1040-FAE. The hydrolytic reactions contained 50 nM of enzyme (Bi1040-FAE) with 200 µM of the respective feruloylated oligosaccharide (with structure shown). **a** Incubated with FAAA (ferulic acid linked to arabinotriose). **b** Incubated with FGG (ferulic acid linked to cellobiose or disaccharide of glucose). **c** Incubated with FAXG (ferulic acid linked to a trisaccharide of arabinose, xylose and glucose). **d** Incubated with InWAX (insoluble wheat arabinoxylan). All reactions were carried out for 10 minutes at 37 °C in a buffer of pH 6.5. The end products were analyzed by C18-HPLC, as described in the Methods section. Fa: ferulic acid. The y-axis is absorbance in milli-absorbance units (mAU). The results are presented as the mean ± the standard deviation of three independent reactions (n=3), and the traces are representatives of the three different reactions. The source data underlying Supplementary Fig. 3 are provided in the Source Data file.

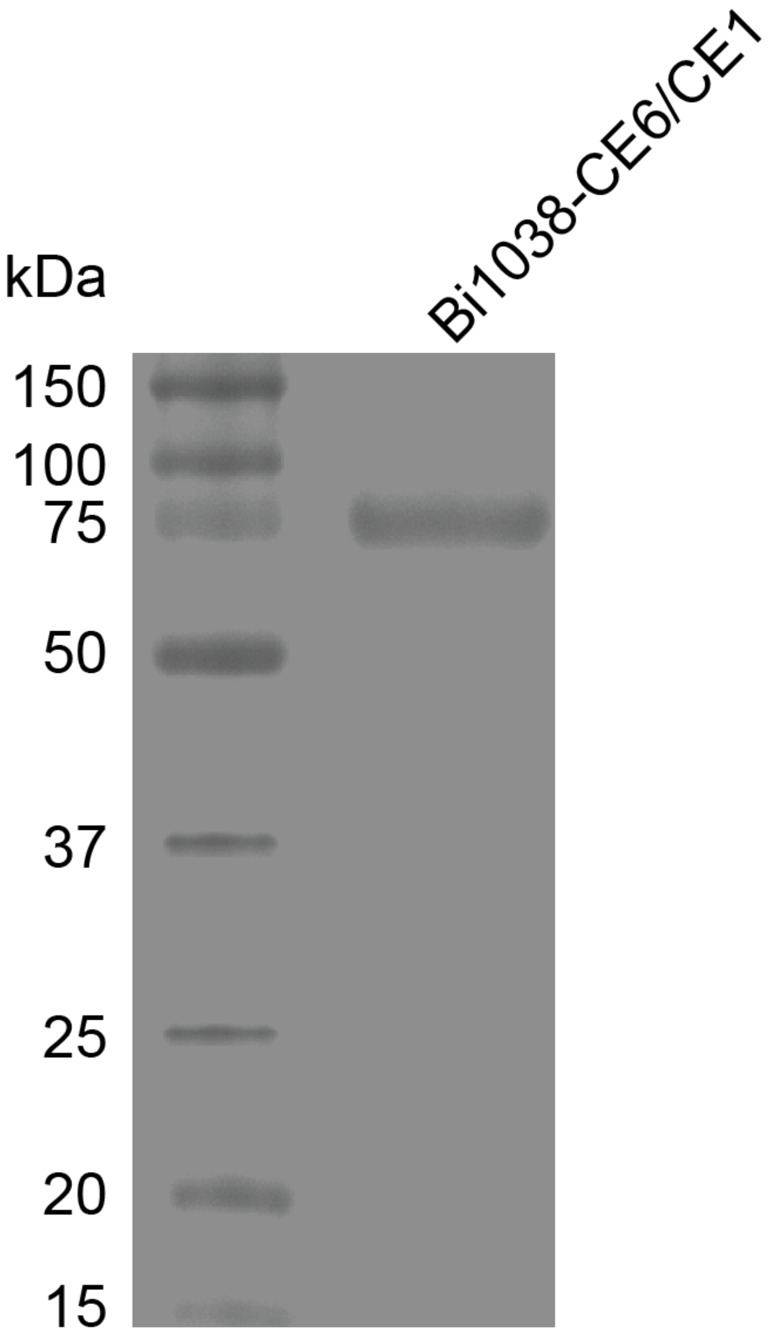

Supplementary Fig. 4. The serine protease inhibitor, benzamidine HCl, inhibits cleavage of recombinant Bi1038-CE6/CE1 during purification from *E. coli* cells. A 12% SDS-PAGE showing purified full length Bi1038-CE6/CE1, achievable by adding benzamidine HCl at a concentration of 1 mM to the buffers for re-suspension of recombinant *E. coli* cells and buffers for protein purification. The recombinant protein was resolved on SDS-PAGE during the purification steps to ensure that it migrates according to its predicted molecular mass, and finally a single SDS-PAGE was ran to obtain this image showing migration to the same position relative to the protein molecular mass markers.

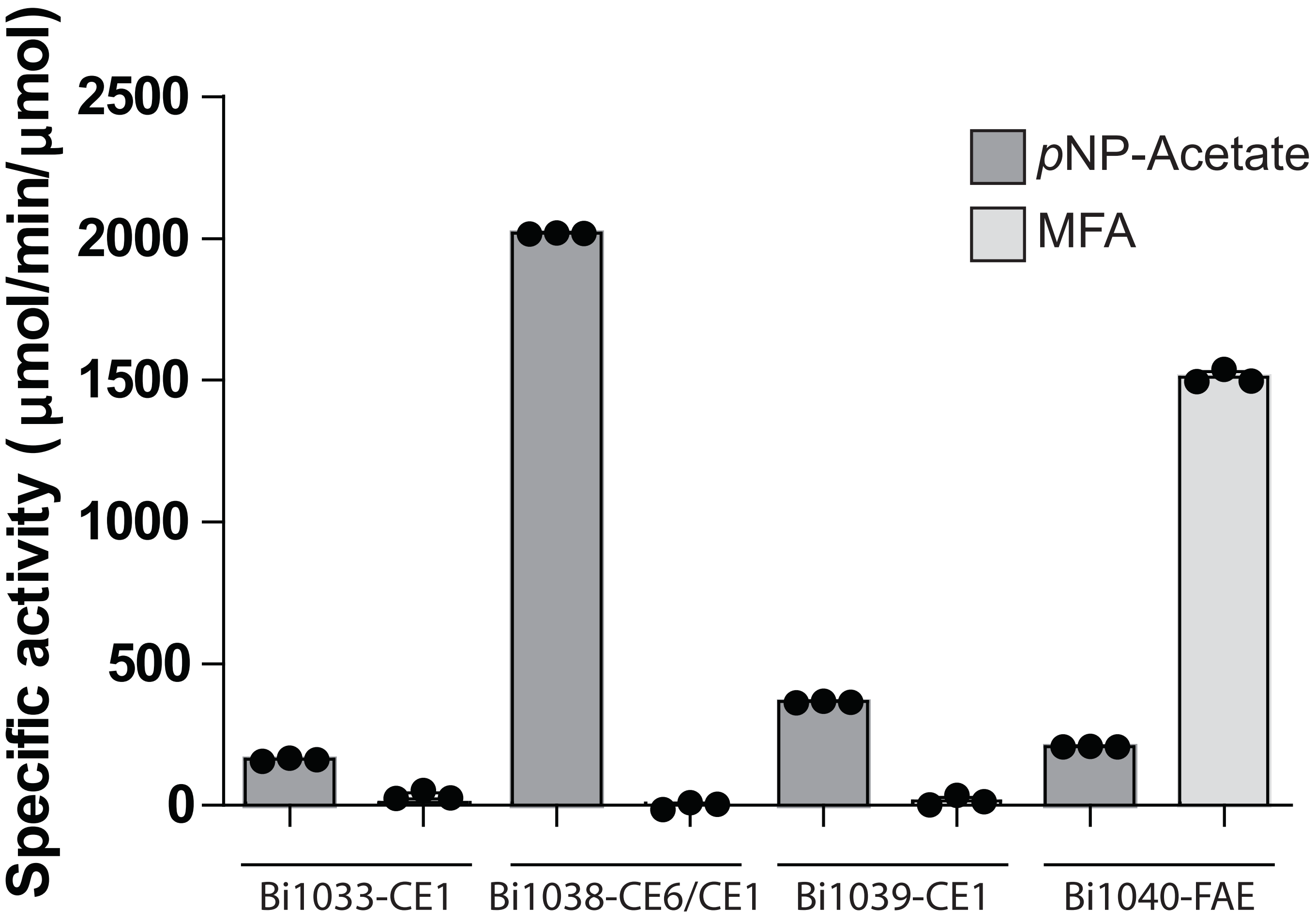

Supplementary Fig. 5. Hydrolytic activities of the recombinant esterases from the *B. intestinalis* EGE PUL. The two synthetic substrates methyl ferulate (MFA) and *para*-nitrophenyl acetate (*p*-NP Acetate) were used to analyze for ferulic acid esterase and acetyl xylan esterase activities, respectively, among the recombinant esterases expressed from the *B. intestinalis* EGE PUL. The reactions were carried out in a 0.1 M MOPS buffer at pH 7.5 and at a temperature of 37 °C. The results are the mean specific activity ± standard deviation of three independent reactions (n = 3). The source data underlying Supplementary Fig. 5 are provided in the Source Data file.

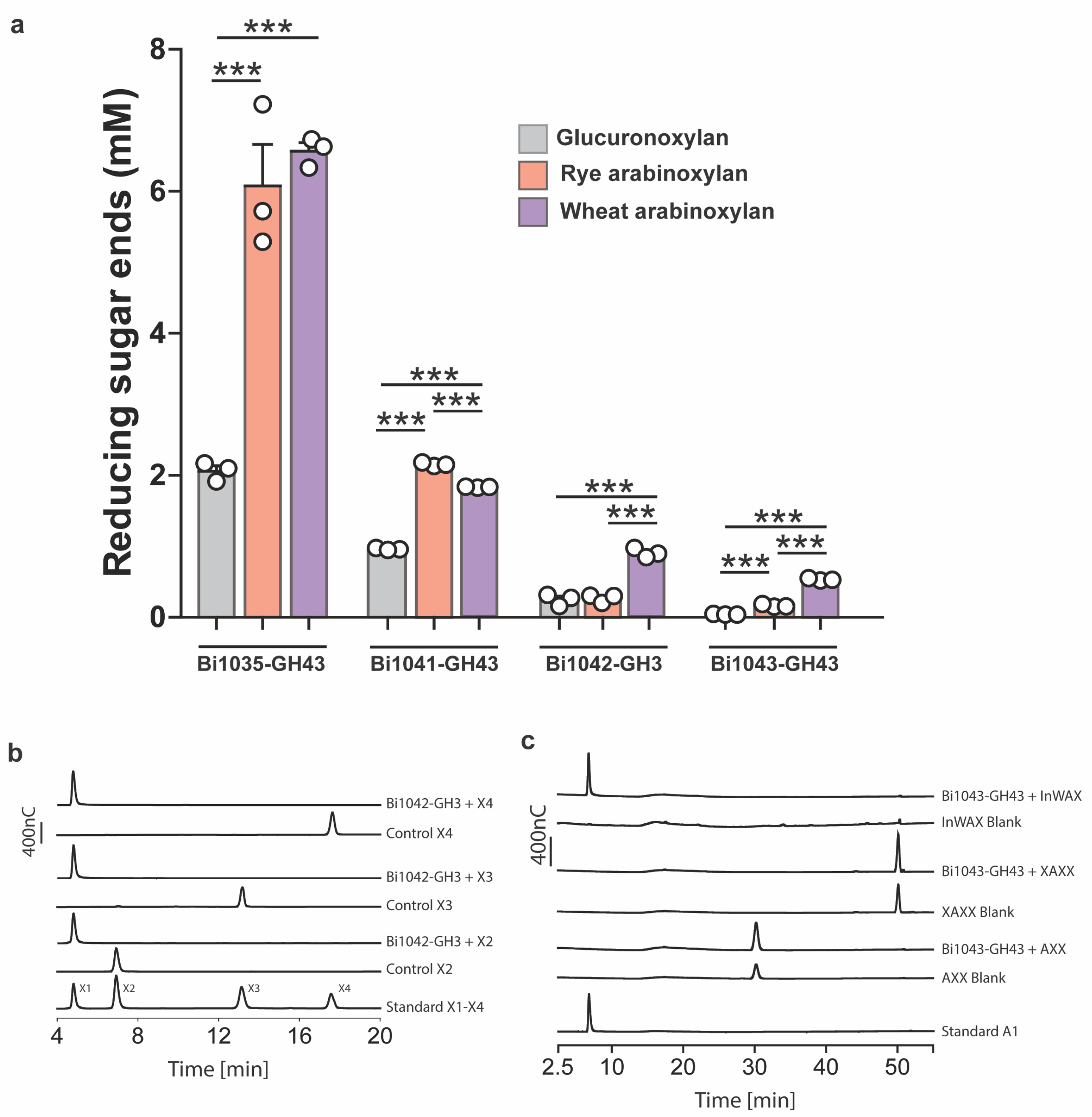
a
Reducing sugar ends (mM)
8
6
4
2
0
***
Glucuronoxylan
Rye arabinoxylan
Wheat arabinoxylan
Bi1035-GH43
Bi1041-GH43
Bi1042-GH3
Bi1043-GH43
b
400nC
Bi1042-GH3 + X4
Control X4
Bi1042-GH3 + X3
Control X3
Bi1042-GH3 + X2
Control X2
X1
X2
X3
X4
Standard X1-X4
4
8
12
16
20
Time [min]
c
400nC
Bi1043-GH43 + InWAX
InWAX Blank
Bi1043-GH43 + XAXX
XAXX Blank
Bi1043-GH43 + AXX
AXX Blank
Standard A1
2.5
10
20
30
40
50
Time [min]

Supplementary Fig 6. Hydrolytic activities of the glycoside hydrolases encoded in the EGE PUL of *B. intestinalis*. a The reducing ends released from xylan polysaccharide substrates by the putative glycoside hydrolases in the *B. intestinalis* EGE PUL. **b** β-xylosidase activity of Bi1042-GH3 towards xylo-oligosaccharides. **c** Arabinoxylan polysaccharide-dependent α-arabinofuranosidase activity of Bi1043-GH43. Rye arabinoxylan (Megazyme) has a backbone of xylose linked in β-1,4 glycosidic linkages and arabinose side chains at the O-2 or O-3 positions or both. The ratio of arabinose to xylose is 40:60. Glucuronoxylan (Sigma) has a backbone of xylose linked in β-1,4 glycosidic linkages and glucuronate side chains at the O-2 or O-3 positions or both, which are commonly methylated at position 4. Wheat arabinoxylan (Megazyme) has a backbone of xylose linked in β-1,4 glycosidic linkages and arabinose side chains at the O-2 or O-3 positions or both. The ratio of arabinose to xylose is 38:62. X1: xylose, X2: xylobiose, X3: xylotriose, X4: xylotetraose, AXX: a trisaccharide of arabinose and two xylose residues, XAXX: a tetra-saccharide of xylose, arabinose and two xylose residues, and InWAX: insoluble wheat arabinoxylan (see Supplementary Fig. 1b). In **a**, the bars indicate means ± standard deviation of three independent reactions (n=3). Statistical analysis was performed using one-way Analysis of Variance (ANOVA) with multiple comparisons using Tukey’s test, *$p<0.033$, **$p<0.002$, ***$p<0.001$. The experiments reported in **b** and **c** were carried out in single reactions. The source data underlying Supplementary Fig. 6a are provided in the Source Data file.

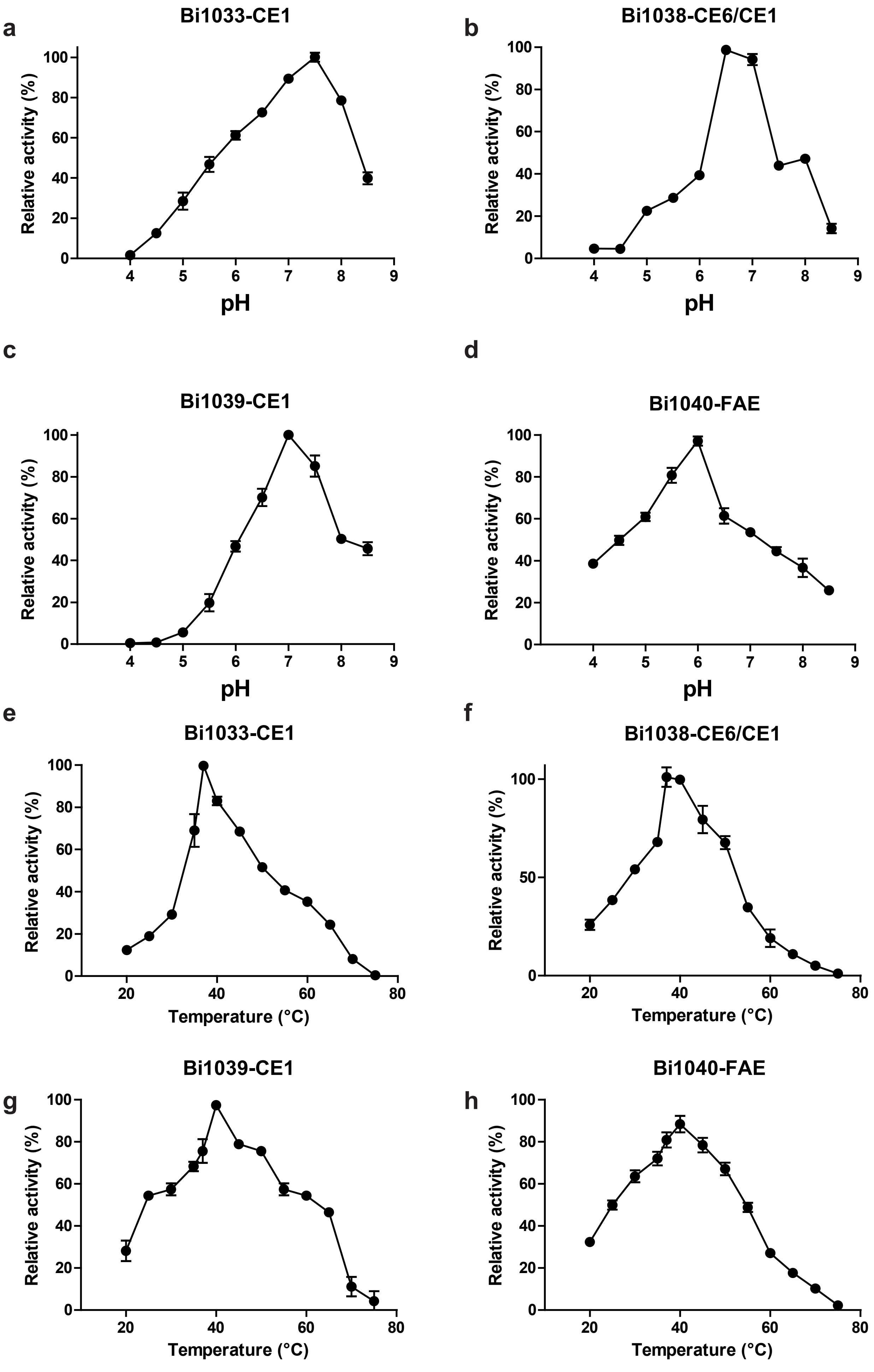
a
Bi1033-CE1
Relative activity (%)
0
20
40
60
80
100
4
5
6
7
8
9
pH
b
Bi1038-CE6/CE1
Relative activity (%)
0
20
40
60
80
100
4
5
6
7
8
9
pH
c
Bi1039-CE1
Relative activity (%)
0
20
40
60
80
100
4
5
6
7
8
9
pH
d
Bi1040-FAE
Relative activity (%)
0
20
40
60
80
100
4
5
6
7
8
9
pH
e
Bi1033-CE1
Relative activity (%)
0
20
40
60
80
100
20
40
60
80
Temperature (°C)
f
Bi1038-CE6/CE1
Relative activity (%)
0
50
100
20
40
60
80
Temperature (°C)
g
Bi1039-CE1
Relative activity (%)
0
20
40
60
80
100
20
40
60
80
Temperature (°C)
h
Bi1040-FAE
Relative activity (%)
0
20
40
60
80
100
20
40
60
80
Temperature (°C)

Supplementary Fig. 7. Determination of the pH and temperature optima of the recombinant esterases from the *B. intestinalis* EGE PUL. The enzymatic activity of each esterase was determined at different pH at 37 °C using two different buffers and *p*NP-acetate as substrate. The relative activities at different pH were then calculated as the percent of the highest obtained activity (**a, b, c,** and **d**). The optimal temperatures were then determined by incubating each enzyme at its respective optimal pH at temperatures ranging from 20 °C to 75 °C, and the relative activity was calculated relation to the highest activity obtained (**e, f, g,** and **h**). The graphs were plotted from the means ± standard deviation of three independent reactions (n = 3). The source data underlying Supplementary Figs. 7a-h are provided in the Source Data file.

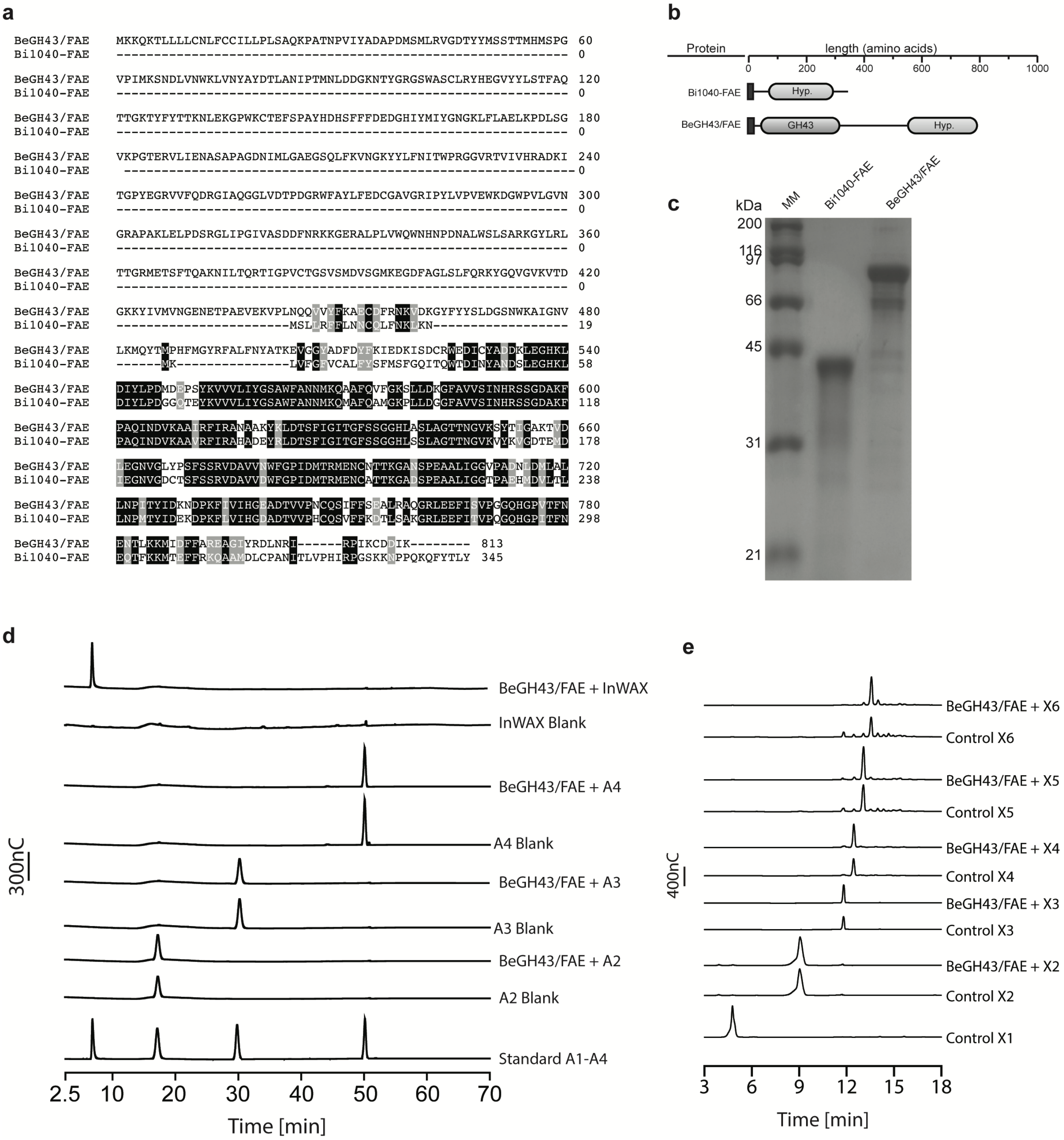
a
BeGH43/FAE MKKQKTLLLLCNLFCCILLPLSAQKPATNPVIYADAPDMSMLRVGDTYYMSSTTMHMSPG 60
Bi1040-FAE -- 0
BeGH43/FAE VPIMKSNDLVNWKLVNYAYDTLANIPTMNLDDGKNTYGRGSWASCLRYHEGVYYLSTFAQ 120
Bi1040-FAE -- 0
BeGH43/FAE TTGKTYFYTTKNLEKGPWKCTEFSPAYHDHSFFFDEDGHIYMIYGNGKLFLAELKPDLSG 180
Bi1040-FAE -- 0
BeGH43/FAE VKPGTERVLIENASAPAGDNIMLGAEGSQLFKVNGKYYLFNITWPRGGVRTVIVHRADKI 240
Bi1040-FAE -- 0
BeGH43/FAE TGPYEGRVVFQDRGIAQGGLVDTPDGRWFAYLLFEDCGAVGRIPYLVPVEWKDGWPVLGVN 300
Bi1040-FAE -- 0
BeGH43/FAE GRAPAKLELPDSRGLIPGIVASDDFNRKKGERALPLVWQWNHNPDNALWSLSARKGYLRL 360
Bi1040-FAE -- 0
BeGH43/FAE TTGRMETSFTQAKNILTQRTIGPVCTGSVSMDVSGMKEGDFAGLSLFQRKYGQVGVKVTD 420
Bi1040-FAE -- 0
BeGH43/FAE GKKYIVMVNGENETPAEVEKVPLNQQVVYFKAECDFRNKVDKGYFYYSLDGSNWKAIGNV 480
Bi1040-FAE -----------------------MSLLRFFLNNCQLFNKLKN------------------ 19
BeGH43/FAE LKMQYTMPHFMGYRFALFNYATKEVGGYADFDYFKIEDKISDCRWEDICYADDKLEGHKL 540
Bi1040-FAE -------MK---------------LVFGFVCALFYSFMSFGQITQWTDINYANDSLEGHKL 58
BeGH43/FAE DIYLPDMDEPSYKVVVLIYGSAWFANNMKQAAFQVFGKSLLDKGFAVVSINHRSSGDAKF 600
Bi1040-FAE DIYLPDGGQTEYKVVVLIYGSAWFANNMKQMAFQAMGKPLLDGGFAVVSINHRSSGDAKF 118
BeGH43/FAE PAQINDVKAAIRFIRANAAKYKLDTSFIGITGFSSGGHLASLAGTTNGVKSYTIGAKTVD 660
Bi1040-FAE PAQINDVKAAVRFIRAHADEYRLDTSFIGITGFSSGGHLSSLAGTTNGVKVYKVGDTEMD 178
BeGH43/FAE LEGNVGLYPSFSSRVDAVVNWFGPIDMTRMENCNTTKGANSPEAALIGGVPADNLDMLAL 720
Bi1040-FAE IEGNVGDCTSFSSRVDAVVDWFGPIDMTRMENCATTKGADSPEAALIGGTPAEHMDVLTL 238
BeGH43/FAE LNPITYIDKNDPKFIVIHGEADTVVPNCQSIFFSEALRAQGRLEEFISVPGGQHGPVTFN 780
Bi1040-FAE LNPMTYIDEKDPKFLVIHGDADTVVPHCQSVFFKDTLSAKGRLEEFITVPQGQHGPITFN 298
BeGH43/FAE ENTLKKMIDFFAREAGIYRDLNRI------RPIKCDDIK-------- 813
Bi1040-FAE EQTFKKMTEFFRKQAAMDLCPANITLVPHIRPGSKKNPPQKQFYTLY 345
b
Protein
length (amino acids)
0
200
400
600
800
1000
Bi1040-FAE
Hyp.
BeGH43/FAE
GH43
Hyp.
c
kDa
MM
Bi1040-FAE
BeGH43/FAE
200
116
97
66
45
31
21
d
BeGH43/FAE + InWAX
InWAX Blank
BeGH43/FAE + A4
A4 Blank
BeGH43/FAE + A3
A3 Blank
BeGH43/FAE + A2
A2 Blank
Standard A1-A4
300nC
2.5
10
20
30
40
50
60
70
Time [min]
e
BeGH43/FAE + X6
Control X6
BeGH43/FAE + X5
Control X5
BeGH43/FAE + X4
Control X4
BeGH43/FAE + X3
Control X3
BeGH43/FAE + X2
Control X2
Control X1
400nC
3
6
9
12
15
18
Time [min]

Supplementary Fig. 8. Comparison of Bi1040-FAE and BeGH43/FAE demonstrate high conservation of the FAE module. a The polypeptide sequence of the hypothetical protein (Bi1040), demonstrated as a ferulic acid esterase (FAE), aligned with BeGH43/Hyp showed high conservation of the C-terminal region of BeGH43/Hyp with Bi1040-FAE leading to designation as BeGH43/FAE. **b** A schematic showing the modular architectures of Bi1040-FAE and BeGH43/FAE. **c** A 12% SDS-PAGE analysis showing purified recombinant Bi1040-FAE and BeGH43/FAE. **d** HPLC chromatograms showing that BeGH43/FAE cleaves arabinose off insoluble arabinoxylan (InWAX) and not from arabino-oligosaccharides (A2, A3 or A4). **e** HPLC chromatograms showing that BeGH43/FAE does not hydrolyze xylo-oligosaccharides (X2, X3, X4, X5, and X6). The experiments in **d** and **e** were from single reactions. InWAX: insoluble wheat arabinoxylan; A2, A3, A4 are straight chain arabinobiose, arabinotriose and arabinotetraose, respectively; X2, X3, X4, X5, X6, xylobiobiose, xylotriose, xylotetraose, xylopentaose, and xylohexaose, respectively.

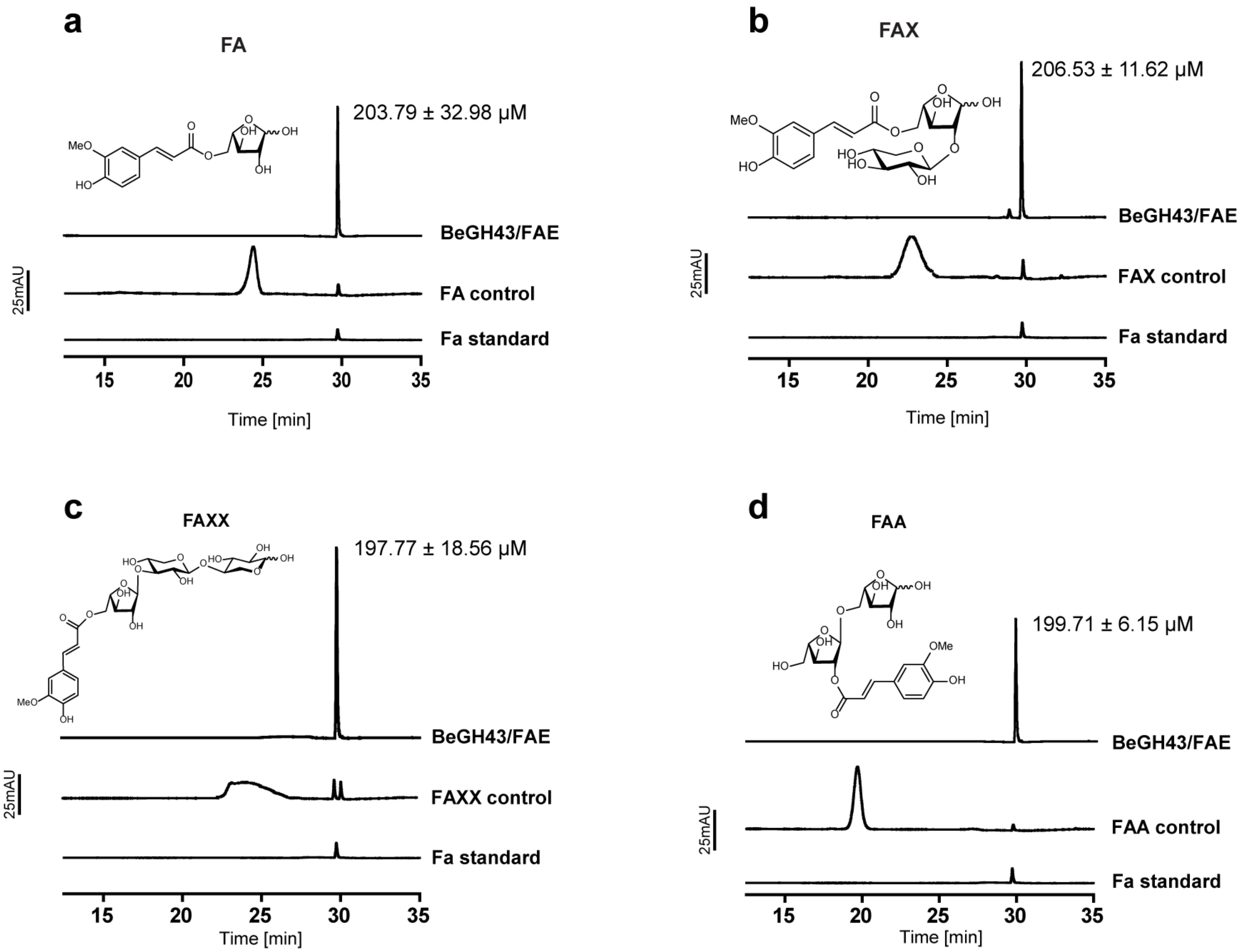

a
FA
203.79 ± 32.98 µM
BeGH43/FAE
FA control
Fa standard
25mAU
15
20
25
30
35
Time [min]
b
FAX
206.53 ± 11.62 µM
BeGH43/FAE
FAX control
Fa standard
25mAU
15
20
25
30
35
Time [min]
c
FAXX
197.77 ± 18.56 µM
BeGH43/FAE
FAXX control
Fa standard
25mAU
15
20
25
30
35
Time [min]
d
FAA
199.71 ± 6.15 µM
BeGH43/FAE
FAA control
Fa standard
25mAU
15
20
25
30
35
Time [min]

Supplementary Fig. 9. Cleavage of ferulic acid from feruloylated oligosaccharides by BeGH43/FAE. The hydrolytic reactions contained 50 nM of enzyme (BeGH43/FAE) with 200 μM of the respective feruloylated oligosaccharide (with structure shown). **a** Incubated with FA (ferulic acid linked to arabinose). **b** Incubated with FAX (ferulic acid linked to a disaccharide of arabinose and xylose). **c** Incubated with FAXX (ferulic acid linked to a trisaccharide of arabinose and two xylose residues). **d** Incubated with FAA (ferulic acid linked to arabinobiose). All reactions were carried out for 10 minutes at 37 °C in a buffer of pH 6.5. The end products were analyzed by C18-HPLC, as described in the Methods section. Fa: ferulic acid. The y-axis is absorbance in milli-absorbance units (mAU). The results are presented as the mean ± the standard deviation of three independent reactions (n=3), and the traces are representatives of the three different reactions. The source data underlying Supplementary Fig. 9 are provided in the Source Data file.

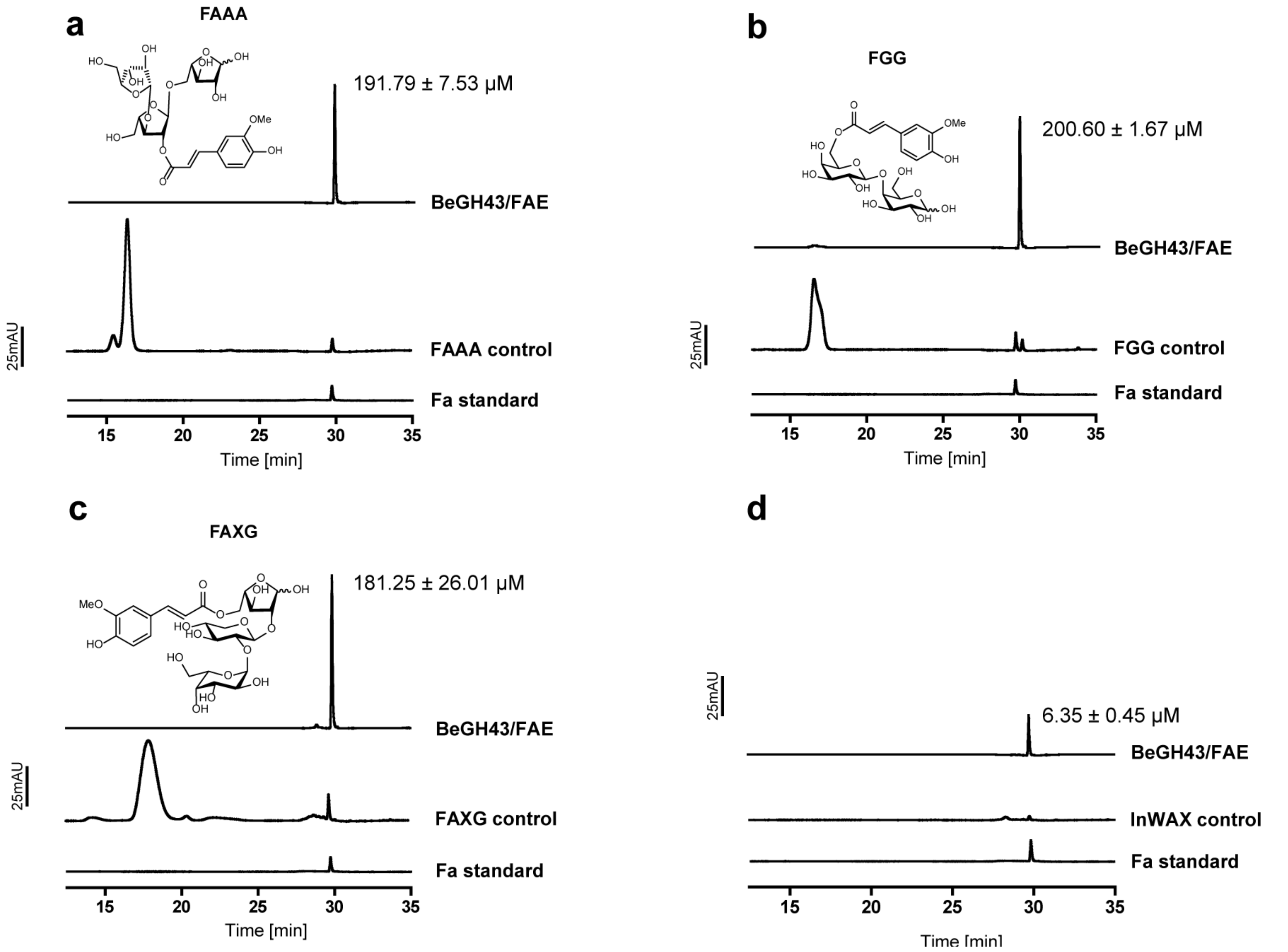

a
FAAA
191.79 ± 7.53 μM
BeGH43/FAE
FAAA control
Fa standard
25mAU
Time [min]
b
FGG
200.60 ± 1.67 μM
BeGH43/FAE
FGG control
Fa standard
25mAU
Time [min]
c
FAXG
181.25 ± 26.01 μM
BeGH43/FAE
FAXG control
Fa standard
25mAU
Time [min]
d
6.35 ± 0.45 μM
BeGH43/FAE
InWAX control
Fa standard
25mAU
Time [min]

Supplementary Fig. 10. Cleavage of ferulic acid from feruloylated oligosaccharides and InWAX by BeGH43/FAE. The hydrolytic reactions contained 50 nM of enzyme (BeGH43/FAE) with 200 µM of the respective feruloylated oligosaccharide (with structure shown). **a** Incubated with FAAA (ferulic acid linked to arabinotriose). **b** Incubated with FGG (ferulic acid linked to cellobiose or disaccharide of glucose). **c** Incubated with FAXG (ferulic acid linked to a trisaccharide of arabinose, xylose and glucose). **d** Incubated with InWAX (insoluble wheat arabinoxylan). All reactions were carried out for 10 minutes at 37 °C in a buffer of pH 6.5. The end products were analyzed by C18-HPLC, as described in the Methods section. Fa: ferulic acid. The y-axis is absorbance in milli-absorbance units (mAU). The results are presented as the mean ± the standard deviation of three independent reactions (n=3), and the traces are representatives of the three different reactions. The source data underlying Supplementary Fig. 10 are provided in the Source Data file.

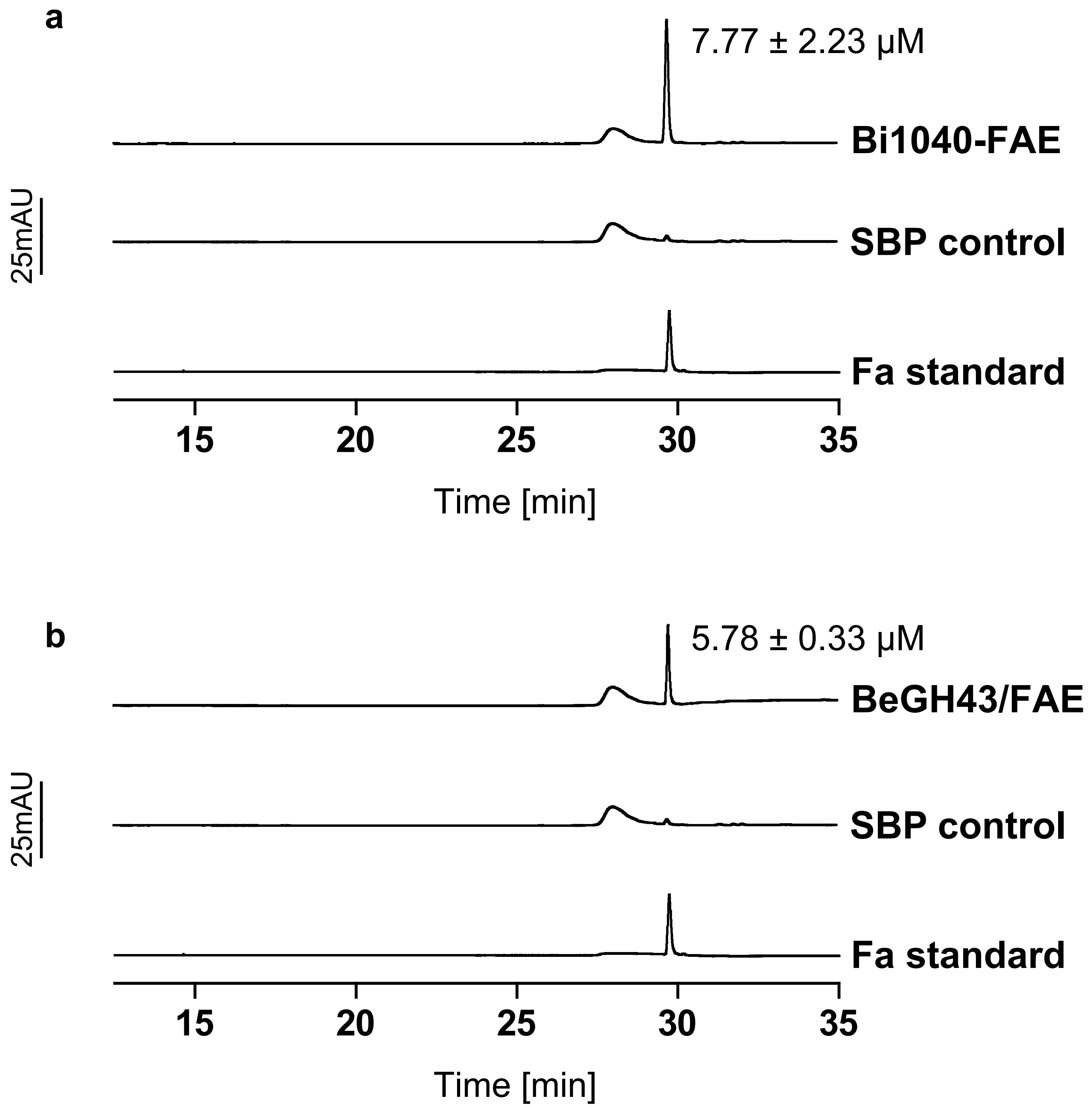

a
7.77 ± 2.23 µM
Bi1040-FAE
25mAU
SBP control
Fa standard
15
20
25
30
35
Time [min]
b
5.78 ± 0.33 µM
BeGH43/FAE
25mAU
SBP control
Fa standard
15
20
25
30
35
Time [min]

Supplementary Fig. 11. Cleavage of ferulic acid from a natural substrate (sugar beet pulp). In substrates such as wheat bran, the ferulic acid is esterified to the C-5 position of arabinose in the arabinoxylan, a β-1,4-D-xylan to which α-L-arabinofuranosyl residues attached at position 2 or 3. By contrast, the feruloyl groups in sugar beet pulp are linked to the arabinofuranosyl residues of the main core of α-1,5 linked arabinan chains and to the galactopyranosyl residues of the main core of the β-1,4-linked type I galactan chains[1]. The ferulic acid linkages in sugar beet pulp is therefore more complex than in wheat bran or wheat arabinoxylan. Here, we assessed the versatility of the new ferulic acid esterase for the capacity to release ferulic acid from the more complex substrate sugar beet pulp. The reaction was carried out by incubating 50 nM of Bi1040-FAE or BeGH43/FAE with 0.5% of a naturally occurring substrate, sugar beet pulp, for 2 hours at 37 °C in a buffer of pH 6.5. The end products of hydrolysis were analyzed by C18-HPLC. **a** ferulic acid release by Bi1040-FAE. **b** Ferulic acid release by BeGH43/FAE. Fa: ferulic acid. SBP: sugar beet pulp. The y-axis is absorbance in milli-absorbance units (mAU). The results are presented as the mean ± the standard deviation of three independent reactions (n=3), and the traces are representatives of the three different reactions. The source data underlying Supplementary Fig. 11 are provided in the Source Data file.

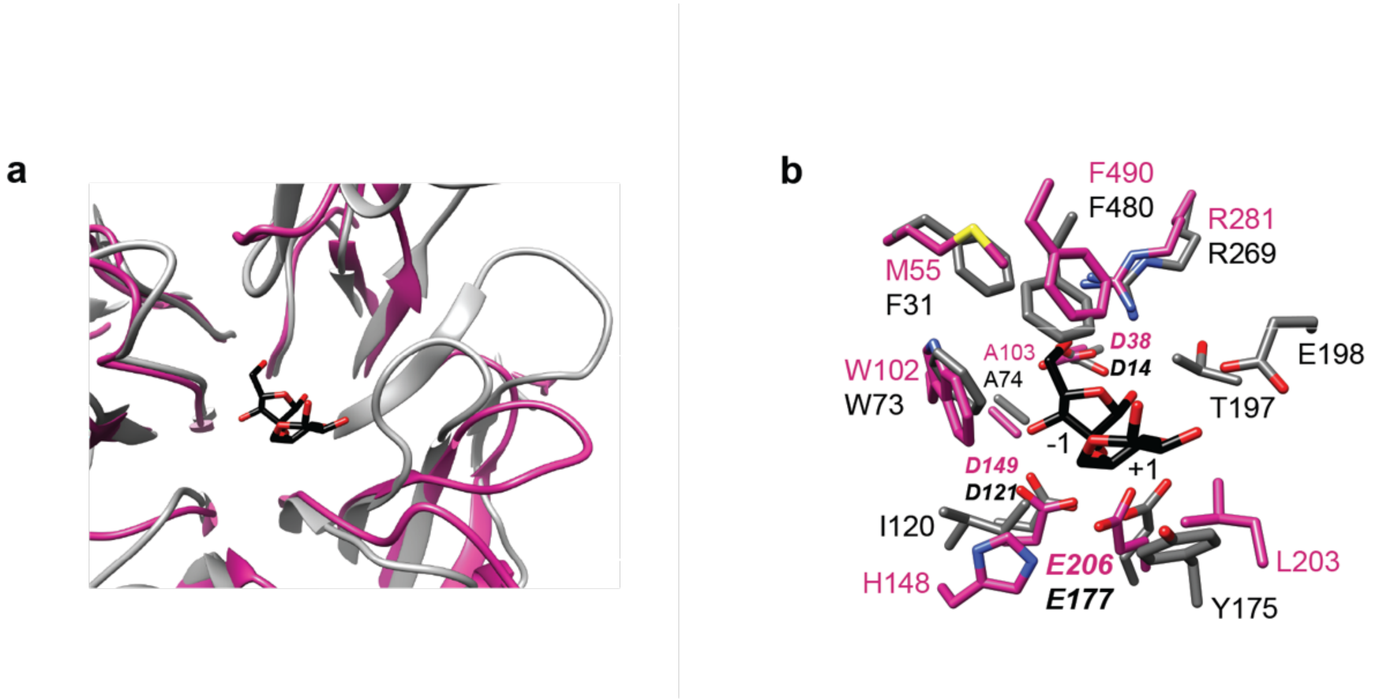

Supplementary Fig. 12. Overlay of the dual function BeGH43/FAE N-terminal domain with the β1,4-xylosidase (GH43) from *Geobacillus thermoleovorans*. a Close up view of the overlay of the N-terminus of the BeGH43/FAE bifunctional enzyme (pink ribbon) with the structure of the β1,4-xylosidase (GH43) from *Geobacillus thermoleovorans* Xyl enzyme (grey, PDB 5z5i, RMSD= 2.1Å for 454 residues) with bound arabinose and xylose. **b** Close up view of the residues coordinating arabinose (-1 subsite) and xylose (+1 subsite) in the *G. thermoleovorans* Xyl structure (grey and black). Residues within 3.5Å of the substrate are displayed for both structures.

a

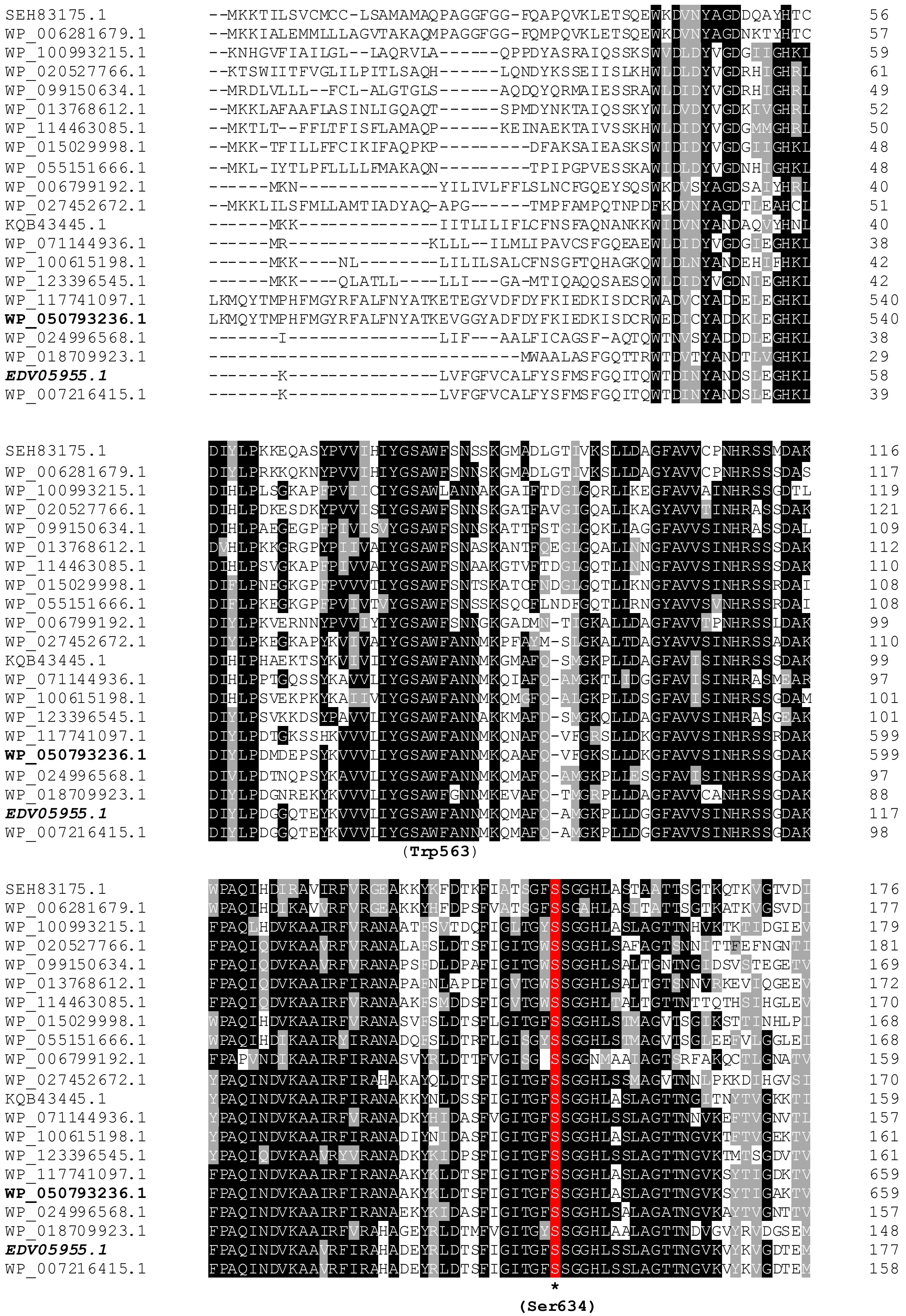
SEH83175.1
WP_006281679.1
WP_100993215.1
WP_020527766.1
WP_099150634.1
WP_013768612.1
WP_114463085.1
WP_015029998.1
WP_055151666.1
WP_006799192.1
WP_027452672.1
KQB43445.1
WP_071144936.1
WP_100615198.1
WP_123396545.1
WP_117741097.1
WP_050793236.1
WP_024996568.1
WP_018709923.1
EDV05955.1
WP_007216415.1
(Trp563)
(Ser634)

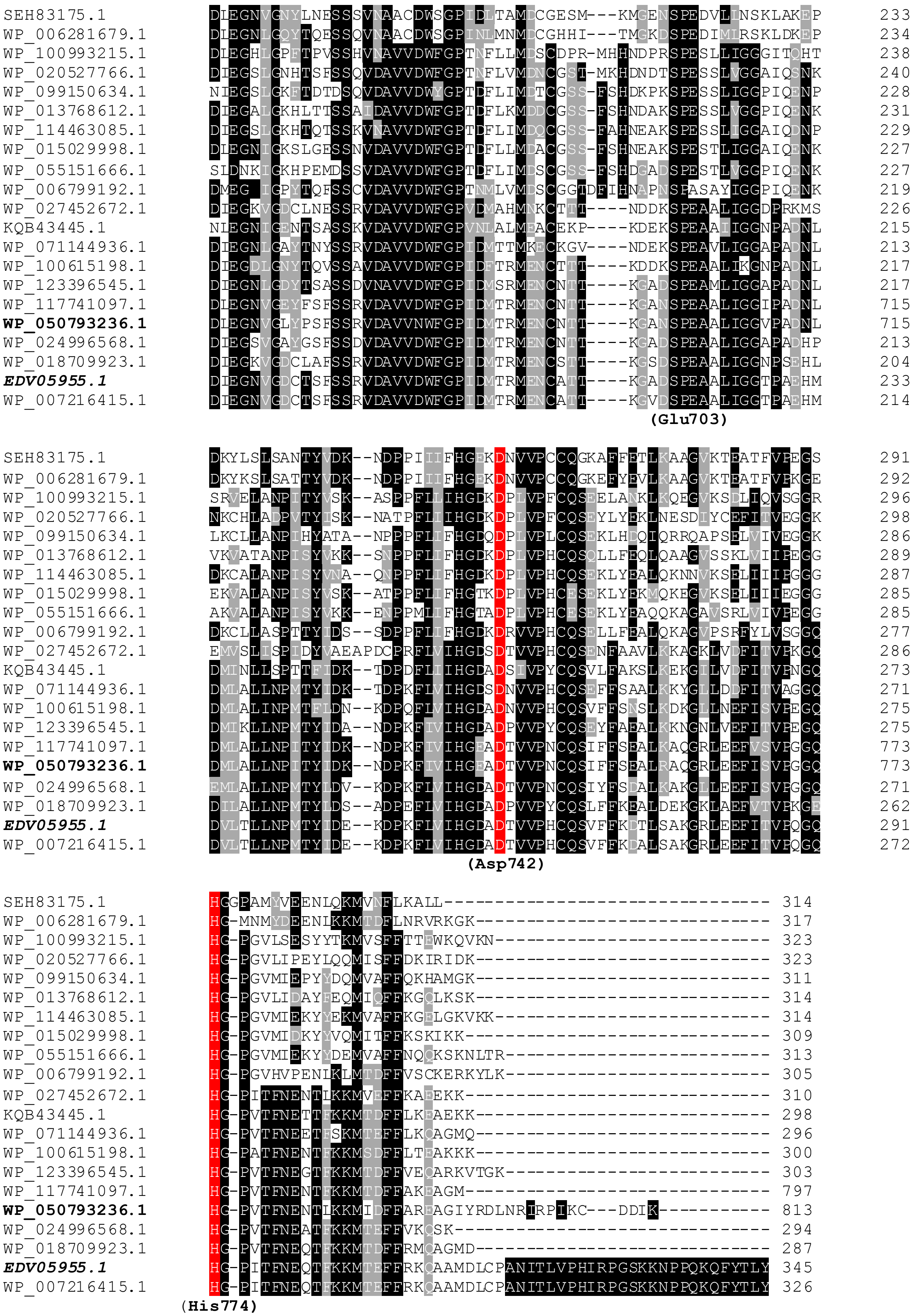

SEH83175.1
WP_006281679.1
WP_100993215.1
WP_020527766.1
WP_099150634.1
WP_013768612.1
WP_114463085.1
WP_015029998.1
WP_055151666.1
WP_006799192.1
WP_027452672.1
KQB43445.1
WP_071144936.1
WP_100615198.1
WP_123396545.1
WP_117741097.1
WP_050793236.1
WP_024996568.1
WP_018709923.1
EDV05955.1
WP_007216415.1
(Glu703)
(Asp742)
(His774)

b

```
PZX17224.1        ----------MIVNNLKILSM--AIAMVMGNITFAQTAKVNDDFKPSEVNQPGKLYPQVN   48
WP_117954567.1    ------------MAMKRIITLFGAF-ALLTNLNSFSYAQALEDFKPSSVNQIGKAYPQVN   47
WP_007661004.1    --------------MKKQILFWSMM-SWMVSVGLPSFAQTVEDFKPSEVNQPGKLYPQVN   45
WP_007216414.1    --------------MKKQILFWSMM-SWMVSVGLPSFAQTVEDFKPSEVNQPGKLYPQVN   45
WP_123120797.1    ----------MNY--KRFIFFAATFLLGASCFA------QSDGFKPSATNAPGKQYPQVS   42
WP_066034169.1    --------MRLSIN--AVIAIFS---LYFQSVNAQSEQIITEDFKPSAANQPERIFPQVN   47
WP_055090457.1    ----------MNYK--RIIYLTFV-CSVTISLAQTKTQEVLEDFKPSSVNQQGKLFPQVN   47
WP_035660712.1    ----------MNFK--SILFSVNV-LASIICTAQTNQIKVIEDFKPSSVNQQGKLYPQVN   47
WP_083552732.1    ----------MNYK--SIIYLVSI-MTTTIGIGQTNPLKVTEDFKPSSVNQQGKLYPQVN   47
WP_066330097.1    ----------MKVN--SIIVFVIT-IISNTSFSQSVSSNVVEDFVPSSVNQQGKLYPQVN   47
WP_068704550.1    ----------MNIKHIFIAFVAA------AFCQMAPAQTVVEDFKPSSVNQPGKMFPQVN   44
WP_020531203.1    MDFLKNLNMTSKINHRYILFLI---GFMLTGVICFAQSEVVEDFKPSSVNQPGKQFPQVN   57
WP_016778581.1    --------------MKYIQSLLVIFALFIGSIATAQTDKVVEDFKPSSVNQQGKEFPQVN   46
WP_106153941.1    --------------MRYIHLLIILF-LLSSNFVTAQTEKVKEDFKPSSVNQIGKEFPQVN   45
WP_053183372.1    --------------MKHFLLILALFVLSATNLVLAQTSKAIEDFKPSEVNQPGKAFPQVN   46
WP_045027037.1    -------------MKNTFVLIAALFICG-----ICTAQDVVEDFKPSSVNQPGKQYPQVN   42
WP_038555069.1    -------------MKNIVILIAALFISG-----ICAAQDVVEDFQPSSVNQPGKQFPQVN   42

PZX17224.1        SQGCVRVQISAPDAKMVQLDIGGVTNNLVKDGNGLWTGESAPQDPGFHYYQLNVDGASVP   108
WP_117954567.1    SEGRVRAQLYAPEAKKVQLDIGGVKYDMIKNEQGFWTGESERQQEGFHYYQLNVDGASVP   107
WP_007661004.1    SERKVRVQISAPEAKVVQLDLGGVKYDLTKDEKGVWTGESAPQQEGFHYYQLNVDGAAVP   105
WP_007216414.1    SERKVRVQISAPEAKVVQLDLGGVKYDLTKDEKGVWTGESAPQQEGFHYYQLNVDGAAVP   105
WP_123120797.1    ADGRVRASIVAPDAHNVQLDIGGKKYDMVKDEKGVWTGESLPQDEGFHYYQLNIDGVSVP   102
WP_066034169.1    SQRRVRASISAPNASKVQLDIGGVKYDMRKDDKGVWTGESNPQDEGFHYYQLNIDGASVP   107
WP_055090457.1    SQGRVRANILAPEANKVQLDLGGIKYDMVKDAKGIWTGESLPQDEGFHYYQLNIDGASVP   107
WP_035660712.1    SERRVRASILAPQANKVQLDLGGIKYDMVKDEKGIWTGVSEPQDEGFHYYQLNIDGASVP   107
WP_083552732.1    SQGRVRTSILAPQANKVQLDLGGTKYDMVKDDKGIWTGVSEPQDEGFHYYQLNIDGASVP   107
WP_066330097.1    SERRVRASILAPQANKVQLDLGGTKYDMVKDDKGIWTGVSNPQDEGFHYYQLNIDGASVP   107
WP_068704550.1    SEGRVRVSIPAPNAQKVQLDIGGVKYDLKKDDKGVWTGESAPQVEGFHYYQLNVDGASVP   104
WP_020531203.1    SERRVRVSIAAPEANLVQLDIGGVKYDLTKDNNGVWTGESAPQDEGFHYYQLNIDGASVP   117
WP_016778581.1    SEGRVRVRIEAPQAKKVQLDIGAVKYDLTKDENGVWTGESAPQDEGFHYYQLNIDGASVP   106
WP_106153941.1    SEGRVRVRIEAPGANNVQLDIGAVKYDLTKDENGVWTGESSPQDEGFHYYQLNIDGASVP   105
WP_053183372.1    SEGRVRAQISAPEAKLVQLDIGGVKYDMVKDEDGVWTGESAPQDEGFHYYQLNIDGASVP   106
WP_045027037.1    SEGRVRAQILAPEANNVRLDIGGVKYEMVKDENGVWTGESEPQDVGFHYYQLNVDGASVP   102
WP_038555069.1    SEGRVRAQISAPEANNVRLDIGGVNYEMKKDENGVWTGESEPQDVGFHYYQLNIDGASVP   102

PZX17224.1        DPGSRYFYGAGRWGSGIEIPADDSHIFALQDVPHGLVSELNYFSKHIGLMRRCFVYTPPG   168
WP_117954567.1    DPGTKYFYGAGRWGSGIEIPAHDEDFYALKDVPHGLVSELNYYSKITQSWRRCFVYTPAG   167
WP_007661004.1    DPGTIYFYGAGRWGSGIEVPAHDADFYALKDVPHGLLSEMNYYSNLTKAWRRCFVYTPAG   165
WP_007216414.1    DPGTIYFYGAGRWGSGIEVPAHDADFYALKDVPHGLLSEMNYYSNLTKAWRRCFVYTPAG   165
WP_123120797.1    DPGTLYFYGAGRWGSGVEIPASDQDFFSLKDVPHGLVSENIYFSKLTNSWRRCFVYTPPG   162
WP_066034169.1    DPGSLYFYGAGRLGSGIEIPSSDQDFFALKNVPHGLVSENIYFSKLTNSFRRCFIYTPEG   167
WP_055090457.1    DPGTVYFYGAGRLGSGIEIPAHDQDFYALKDVPHGLVSENIYFSKVTNSFRRCFVYTPAE   167
WP_035660712.1    DPGTVYFYGAGRLGSAIEIPAADQEFFAIRDVPHGLVSENIYFSKLTNSFRRCFVYTPAN   167
WP_083552732.1    DPGTVYFYGAGRLGSAIEIPASDSDFYAMKDVPHGLVSENIYFSKLTNSFRRCFVYTPAN   167
WP_066330097.1    DPGTVYFYGAGRLGSAIEIPASDRDFYAMKDVPHGLVSENIYFSKLTNSFRRCFVYTPAN   167
WP_068704550.1    DPGSLYFYGASRWGSAIEIPAFDQDFYAQKDVPHGLVSQKMYFSKVTNSWRRCFVYTPAE   164
WP_020531203.1    DPGSKYFYGAGRWGSGIEIPAHDREFYALKDVPHGLVSERVYFSKITQVWRQCLVYTPPC   177
WP_016778581.1    DPGTRYFYGAGRWGSGIEIPAHDRDFYALKKVPHGLVSENLYYSDITESWRRCFVYTPPG   166
WP_106153941.1    DPGTKYYYGAGRWGSGIEIPAHDRDFYALKKVPHGLVSENLYYSDITESWRRCFVYTPPG   165
WP_053183372.1    DPGTKFFYGAGRWGSGIEIPAHDRDFYAMKEVPHGLVSELNYFSPLTQSFRRCFVYTPPG   166
WP_045027037.1    DPGTKYFYGAGRWGSGIEIPADDRNIYALKDVPHGLVSEQNYFSEITQSFRRCFVYTPAE   162
WP_038555069.1    DPGTNYFYGAGRWGSGIEIPAKDMDTYALKNVPHGLVSEQPYFSEITQSFRRCFVYTPAE   162

PZX17224.1        YEADANMRYPVLYLQHGSFEDETGWIGQGKANLILDNLIAHHKALPMIVVMDNGYAEKPL   228
WP_117954567.1    YEVNTERRYPVLYLQHGSFEDETGWGSQGKANLILDNLIAAKKAVPMLIVMDNGYATRPS   227
WP_007661004.1    YGDNKDKRYPVLYLQHGSFEDETGWGRQGKTNLILDNLIAAGKAVPMLVVMDNGYATKPG   225
WP_007216414.1    YGDNKDKRYPVLYLQHGSFEDETGWGRQGKTNLILDNLIAAGKAVPMLVVMDNGYATKPG   225
WP_123120797.1    YEKDAKTRYPVLYLQHGSFEDETGWSRQGHANLILDNLIASGKAVPMIIVMDNGYASKPS   222
WP_066034169.1    YNENTKTRYPVLYLQHGSFEDETGWSAQGKANLILDNLIASKKAVPMIIVMDNGYAYKAQ   227
WP_055090457.1    YFQNQNKRYPVLYLQHGSFEDETGWSVQGKANLILDNLIAAQKANPMIVVMDNGYAYKPQ   227
WP_035660712.1    YNEDSKTRFPVLYLQHGSFEDETGWAVQGKANLILDNLIASKKSVPMIIVMDNGYAYKPQ   227
WP_083552732.1    YNENTNTRYPVLYLQHGSFEDETGWAVQGKANLILDNLIASKKANPMIVVMDNGYAYKPQ   227
WP_066330097.1    YNTDTKTRFPVLYLQHGSFEDETGWAVQGKANLILDNLITSKKAKPMIVVMDNGYAYKPQ   227
WP_068704550.1    YDKNPSKRYPVLYLQHGSGEDETGWPTQGKANLILDNLIAAKKAVPMIIVMDNGYATKAN   224
WP_020531203.1    YENDNAKRYPVLYLQHGSFEDETGWSAQGHASLILDNLIAEKKAVPMIIVMDNGYAYKPQ   237
WP_016778581.1    YHRDLSKRYPVLYLQHGSFEDETGWSNQGHANLILDNLIAENKAVPMIIVMDNGYAFKPQ   226
WP_106153941.1    YYEDINKRYPVLYLQHGSFEDETGWSSQGHANLILDNLIAEKKAVPMMIVMDNGYAFKAQ   225
WP_053183372.1    YETSTDQRYPVLYLQHGSFEDETGWPGQGKANLILDNLIAEKKAVPMIIVMDNGYAYEPH   226
WP_045027037.1    YTNNPEKRYPVLYLQHGSFEDETGWPGQGQANCILDNLIAANKAVPMIIVMDNGYAYKPQ   222
WP_038555069.1    YNANPKKQYPVLYLQHGSFEDETGWASQGHANRILDNLIAAQKAVPMIIVMDNGYAYKPQ   222
```

```
PZX17224.1        A--SADGNARPASVFEEVMMQEIIPLIDSRFRTVVHRDHRAIAGLSMGANQTMRIAMNHL   286
WP_117954567.1    EQT-----PFQTTVFEEVLMQEVIPMIDEKFRTLPNRESRAIAGLSMGANQTMRIAMNHP   282
WP_007661004.1    EKS-----PFAASIFEEVLMNEVIPMIDAKFRTLSGREDRAIAGLSMGANQTMHIAMNNP   280
WP_007216414.1    EKS-----PFAASIFEEVLMNEVIPMIDAKFRTLSGREDRAIAGLSMGANQTMHIAMNNP   280
WP_123120797.1    DNP-ADSKTRPQPVFEQVMINEIVPMIDGKFRTIANREHRAIAGLSMGANQTMRIIMNHL   281
WP_066034169.1    ESNS-SDKNPKESVFEDVLITEVIPMIDAKFRTIPKRENRAIAGLSMGANQTMRIMMNHL   286
WP_055090457.1    TNG-----ERQESVFEEVVINEIIPMIDAKFRTLANKENRAIAGLSMGANQTMRIMMNNL   282
WP_035660712.1    SND-----SRQESVFEEVVIQEIIPMIDTKFRTIANRENRAIAGLSMGANQTMRIMMNNL   282
WP_083552732.1    SGT-----ARPESVFEDVLINEIIPMIDAKFRTIANRENRAIAGLSMGANQTMRIMMNNL   282
WP_066330097.1    TNG-----ARQESVFEEVVINEIIPMIDAKFRTIANRENRAIAGLSMGANQTMRIMMNNL   282
WP_068704550.1    A----SAPSKSAFPFEEVMINEIIPMIDASFRTLSDREHRAMAGLSMGANQTITITMNNL   280
WP_020531203.1    NLENADRRGRPASAFEEVMITEIIPMIDTEFRTLKDREHRAIAGLSMGANQTMRIIMNNL   297
WP_016778581.1    E--N-GTTGRPAMVFEEVMMQEIIPMIDARFRTLANRENRAIAGLSMGANQTMRIAMNNL   283
WP_106153941.1    D--K-DAKGRPAMVFEEVMMQEIIPMIDARFRTLANREHRAIAGLSMGANQTMRIAMNNL   282
WP_053183372.1    E--NSDGNSRPVSVFEEVMISEIIPMIDARFRTILDRKHRAIAGLSMGANQTMRIIMNNL   284
WP_045027037.1    T--S--GGGRPAMVFEEVMLNEIIPMIDNRFRTIADREHRAIAGLSMGANQTMRICMNNL   278
WP_038555069.1    S--S--GGGRPAMVFEEVMMNEIIPMIDKRFRTIADREHRAIAGLSMGANQTMRICMNNL   278
                                                             (Ser226)

PZX17224.1        DHFAWYGGFSGTANYPSAQPIDVDTFLDGKFKNGSWVNQQLQLLWLGVGTKEEPVFPKSI   346
WP_117954567.1    EAFAYYGGFSGTSNYPSTEPLDADTFLGGKFKDGKAINRQFKSFFLGLGTSEPAPFPGVV   342
WP_007661004.1    GHFAYYGGFSGTSNYPSTEPLDATTFLNGKFKDAKAVNVQFKVFFLGLGTAEPHPFPGVV   340
WP_007216414.1    GHFAYYGGSSGTSNYPSTEPLDATTFLNGKFKDAKAVNAQFKVFFLGLGTAEPHPFPGVV   340
WP_123120797.1    DTFSSLGAFSGTSNYPNTDVINPATFLDGKFNDGAAINKQFKVLFLGLGTKEPNPFPGSV   341
WP_066034169.1    DTFAYYGGFSGTANYPSSDAIDASTFLNGKYKDGNALNKQIKLFWLGLGTKEPAPFPKSV   346
WP_055090457.1    NTFSYYGGFSGTSNYPSADAIDVNTFLDGKYKDGKSVNEKIKLFWLGLGTKEPSPFPGSV   342
WP_035660712.1    DIFSHYGGFSGTSNYPSADAIDVNSFLDGKFKDGETINKKIKLFWLGLGTKEPSPFPGSV   342
WP_083552732.1    DTFSHYGGFSGTSNYPSTDAIDVNTFLDGKFKDGNALNKKVKLFWLGLGTKEPNPFPGSV   342
WP_066330097.1    DTFSHYGGFSGTSNYPSADAIDVNTFLDGKFKDANALNKKIKLFWLGLGTKEPSPFPGSV   342
WP_068704550.1    DKFSYIAGFSGTSNYPRTEAIDVEKFMGGKFKDGAALNKQIKLFWLGLGTVEPAPFPGSV   340
WP_020531203.1    DVFASYGGFSGTSNYPSADDIDVNTFLGGKFKDGEALSKQIKVFWLGLGTKEPDPFPGSV   357
WP_016778581.1    DHFAYYGGFSGTANYPSSDEIDVETFLDGAFADGEKVDEQLKVLWLGLGTKEPEPFPESI   343
WP_106153941.1    DHFAYYGGFSGTANYPSSDEIDVKTFLDGAFADDKKVDEQLKVLWLGLGTKEPDPFPGSI   342
WP_053183372.1    GVFSNYGGFSGTSNYPSSSEIDPATFLDGKFDDGKKVNEQIDVFWLGLGTKEPEPFPGSV   344
WP_045027037.1    DKFAYYGGFSGTSNYPSSDEINVETFLNGAFKNGKSVNDQIKVFWLGLGTKEPEPFPGSV   338
WP_038555069.1    DKFAYYGGFSGTSNYPSSDEINAETFLNGAFKNGKSVNKQMKVFWLGLGTKEPEPFPGSV   338
                                                                (Glu332)

PZX17224.1        GAFRNMLEQQGIRHVFYVSQGTAHEWHTWRRSLHQYAQLVFK--   388
WP_117954567.1    KAFRQMLDKQGIKYTYYESPETAHEWLTWRRALHQYAQLLFQ--   384
WP_007661004.1    KAFRQMMDKQGIKYVYYESPDTAHEWLTWRRALNEFAPLLFK--   382
WP_007216414.1    KAFRQMMDKQGIKYVYYESPDTAHEWLTWRRALNEFAPLLFK--   382
WP_123120797.1    GAFRNMLDKQGIKYVFYQSPATSHEWLTWRRDLKEFAQLLFKK-   384
WP_066034169.1    GAFRTMLEQQGIKYDYYESPETAHEWLTWRRCLHQFASKLFK--   388
WP_055090457.1    GAFRNMLEKQGIKYTYYESAETAHEWLTWRRCLNQFAAKIFK--   384
WP_035660712.1    RAFRKMLEKQSIKYVYYESPETAHEWLTWRRCLNQFASKIFK--   384
WP_083552732.1    GVFRTMLEQQGIKYAYYESPKTAHEWLTWRRCLNQFATQLFK--   384
WP_066330097.1    GAFRTMLEKQGIKYGYYESPETAHEWLSWRRCLNQFAAKLFQ--   384
WP_068704550.1    KAFRNMLEKQGVKYTYYESQGTAHEWLTWRRDLNQFAALLFK--   382
WP_020531203.1    GAFRNMLDNIGIDHVYYESPGTAHEWLTWRRSLKEYAALLFK--   399
WP_016778581.1    GTFINMLKKQGIQYEYYESPETAHEWLTWRRSLYQYAQLLFK--   385
WP_106153941.1    GAFTNMLKKQGIQYEYYESPETAHEWLTWRRSLYQYAQLLFK--   384
WP_053183372.1    GAFRKMLEKQGIEYVYYESPETAHEWQTWRRSLHEYAQRLFKKN   388
WP_045027037.1    GAFRNMLEKQGIDYVYYESPETAHEWQTWRRSLHQYAQLLFK--   380
WP_038555069.1    GAFRNMLEKQGIDYVYYESPRTAHEWQTWRRSLHQYAQLLFK--   380
                                        (His364)
```

c

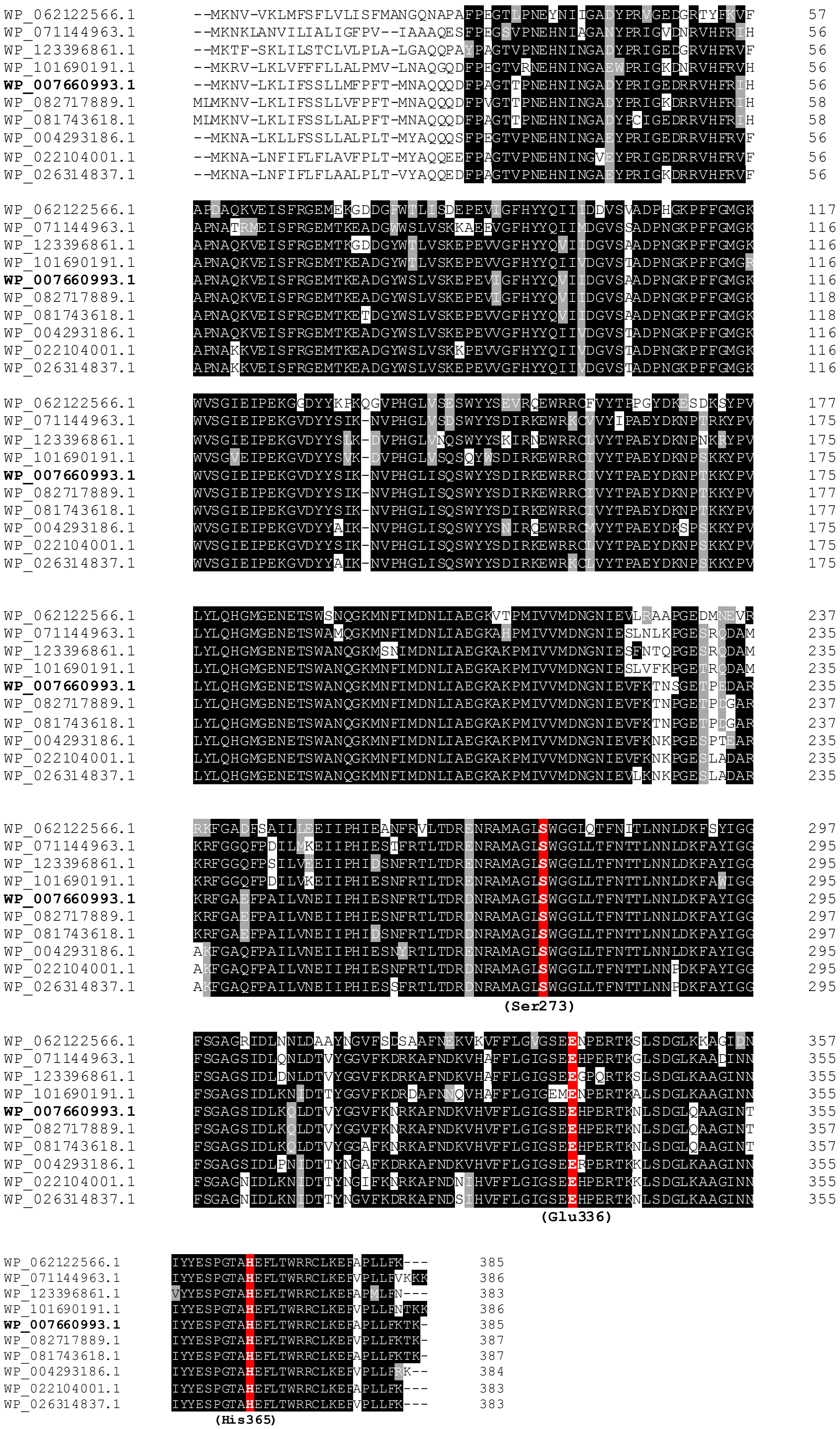

Supplementary Figure 13. Alignments showing that the newly characterized polypeptides associated with the EGE PUL and demonstrated to cleave side-chain ester-linkages from complex arabinoxylans are widely distributed. a A search was made in the Genbank protein database (https://www.ncbi.nlm.nih.gov/protein/) to extract polypeptides with high sequence identities to the *B. intestinalis* homolog (**accession number bolded and italicized-Bi1040-FAE**) and aligned with the CLUSTAL Multiple Alignment program. The Genbank accession numbers and annotations are as follows: SEH83175.1 Acetyl esterase/lipase [*Prevotella ruminicola*]; WP_006281679.1 alpha/beta hydrolase [*Prevotella bryantii*]; >WP_100993215.1 alpha/beta hydrolase [*Spirosoma pollinicola*]; WP_020527766.1 alpha/beta hydrolase [*Flexithrix dorotheae*]; WP_099150634.1 alpha/beta hydrolase [*Lewinella nigricans*]; WP_013768612.1 alpha/beta hydrolase [*Haliscomenobacter hydrossis*]; WP_114463085.1 alpha/beta hydrolase [*Runella* sp. YX9]; WP_015029998.1 alpha/beta hydrolase [*Emticicia oligotrophica*]; WP_055151666.1 alpha/beta hydrolase [*Jiulongibacter sediminis*]; WP_006799192.1 alpha/beta hydrolase [*Dysgonomonas gadei*]; WP_027452672.1 alpha/beta hydrolase [*Prevotella albensis*]; KQB43445.1 Esterase/lipase [*Flavobacterium daejeonense*]; WP_071144936.1 alpha/beta hydrolase [*Bacteroides ihuae*]; WP_100615198.1 alpha/beta hydrolase [*Confluentibacter citreus*]; WP_123396545.1 alpha/beta hydrolase [*Muribaculaceae* bacterium Isolate-102 (HZI)]; WP_117741097.1 glycoside hydrolase [*Bacteroides stercoris*]; >WP_050793236.1 glycoside hydrolase [**bolded**-*Bacteroides eggerthii*]; WP_024996568.1 alpha/beta hydrolase [*Bacteroides graminisolvens*]; >WP_018709923.1 alpha/beta hydrolase [*Bacteroides barnesiae*]; EDV05955.1 hypothetical protein BACINT_01040-FAE [*Bacteroides intestinalis*]; and WP_007216415.1 alpha/beta hydrolase [*Bacteroides cellulosilyticus*]. The *B. eggerthii* and *B. stercoris* polypeptides are from the C-terminal esterase module of the bi-functional enzyme (GH43/FAE). The crystal structure of the *B. eggerthii* bi-functional enzyme (bolded) was solved in this study. **b** A search was made in the Genbank protein database to extract polypeptides with high sequence identities to the *B. intestinalis* homolog (**accession number bolded-Bi1039-CE1**) and aligned with the CLUSTAL Multiple Alignment program. The Genbank accession numbers and annotations are as follows: PZX17224.1 enterochelin esterase family protein [*Cytophaga xylanolytica*]; WP_117954567.1 esterase [*Bacteroides stercoris*]; WP_007661004.1 esterase [*Bacteroides intestinalis*]; WP_007216414.1 esterase [*Bacteroides cellulosilyticus*]; WP_123120797.1 esterase [*Ferruginibacter* sp. BO-59]; WP_066034169.1 esterase [*Flavobacterium anhuiense*]; WP_055090457.1 esterase [*Flavobacterium daejeonense*]; WP_035660712.1 esterase [*Flavobacterium seoulense*]; WP_083552732.1 esterase [*Flavobacterium flevense*]; WP_066330097.1 esterase [*Flavobacterium glycines*]; WP_068704550.1 esterase [*Paludibacter jiangxiensis*]; WP_020531203.1 hypothetical protein

[*Flexithrix dorotheae*]; WP_016778581.1 esterase [*Anaerophaga thermohalophila*]; WP_106153941.1 esterase [*Marinilabilia salmonicolor*]; WP_053183372.1 esterase [*Sunxiuqinia dokdonensis*]; WP_045027037.1 esterase [*Draconibacterium sediminis*]; and WP_038555069.1 esterase [*Draconibacterium orientale*]. The accession number of the *B. intestinalis* enzyme with the crystal structure solved in this study is bolded. **c** A search was made in the Genbank protein database to extract polypeptides with high sequence identities to the *B. intestinalis* homolog (**accession number bolded-Bi1033-CE1**) and aligned with the CLUSTAL Multiple Alignment program. The Genbank Accession numbers and their annotations are as follows: WP_062122566.1 esterase [*Geofilum rubicundum*]; WP_071144963.1 esterase [*Bacteroides ihuae*]; WP_004293186.1 carbohydrate-binding protein [*Bacteroides eggerthii*]; WP_101690191.1 esterase [*Dysgonomonas* sp. Marseille-P4356]; WP_007660993.1 hypothetical protein [*Bacteroides intestinalis*, Bi1033-CE1]; WP_082717889.1 esterase [*Bacteroides cellulosilyticus*]; WP_081743618.1 esterase [*Bacteroides timonensis*]; WP_123396861.1 esterase [Muribaculaceae bacterium Isolate-102 (HZI)]; WP_022104001.1 carbohydrate-binding protein [*Bacteroides stercoris*]; and WP_026314837.1 esterase [*Bacteroides gallinarum*]. The accession number of the *B. intestinalis* enzyme with the crystal structure solved in this study is bolded. The amino acids that identical (shaded black) or similar (grey) in >50% of the positions are indicated. Amino acids with similar properties are grouped as LIMV, AG, YWF, DEQN, KRH and ST. The catalytic triad in each enzyme family are highlighted in red. The N-terminal regions are not shaded, as these regions represent signal peptides for secretion and are unlikely to be involved in the folding of the functional proteins.

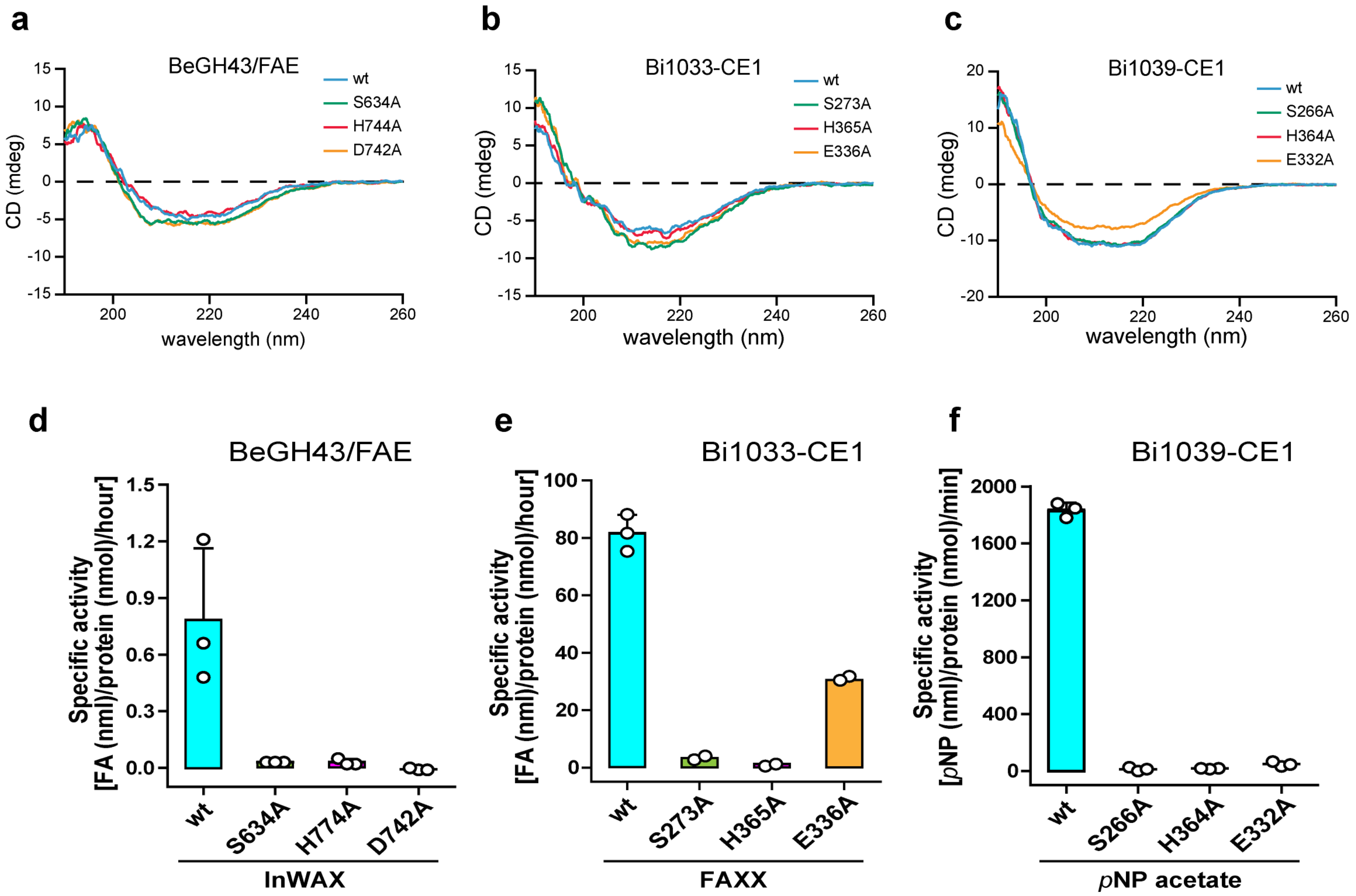
a
BeGH43/FAE
wt
S634A
H744A
D742A
CD (mdeg)
wavelength (nm)
b
Bi1033-CE1
wt
S273A
H365A
E336A
CD (mdeg)
wavelength (nm)
c
Bi1039-CE1
wt
S266A
H364A
E332A
CD (mdeg)
wavelength (nm)
d
BeGH43/FAE
Specific activity
[FA (nml)/protein (nmol)/hour]
wt
S634A
H774A
D742A
InWAX
e
Bi1033-CE1
Specific activity
[FA (nml)/protein (nmol)/hour]
wt
S273A
H365A
E336A
FAXX
f
Bi1039-CE1
Specific activity
[pNP (nml)/protein (nmol)/min]
wt
S266A
H364A
E332A
pNP acetate

Supplementary Fig. 14. **Mutational analysis of the putative catalytic triads.** The residues (Ser-His-Glu/Asp) that constitute the proposed catalytic triad in BeGH43-Fae (S634, H774, D742), Bi1033-CE1 (S273, H365, E336) and Bi1039-CE1 (S266, H364, E332) were substituted with alanine by site-directed mutagenesis. Circular dichroism scans of mutant recombinant proteins of **a** BeGH43/FAE **b** Bi1033-CE1 and **c** Bi1039-CE1 compared to their wild-type counterparts. Proteins (0.1 mg/ml) were dialyzed in 10 mM potassium phosphate buffer pH 7.5 and CD spectra were collected at 25°C from 190 nm to 260 nm at a speed of 50 nm/min with a 0.1-nm wavelength pitch, with five accumulations. Relative feruloyl esterase activity of the mutant **d** BeGH43/FAE **e** Bi1033-CE1 and **f** Bi1039-CE1 proteins presented as a percentage of the activity of the corresponding wild-type proteins on insoluble wheat arabinoxylan (InWAX), the feruloylated trisaccharide (FAXX), and the synthetic substrate, para-nitrophenyl acetate (*p*NP Acetate), respectively. The bars are means ± standard deviations. In **d** and **f**, n = 3 independent reactions. In **e**, n= 2 independent reactions. The source data underlying Supplementary Figs. 14d-f are provided in the Source Data file.

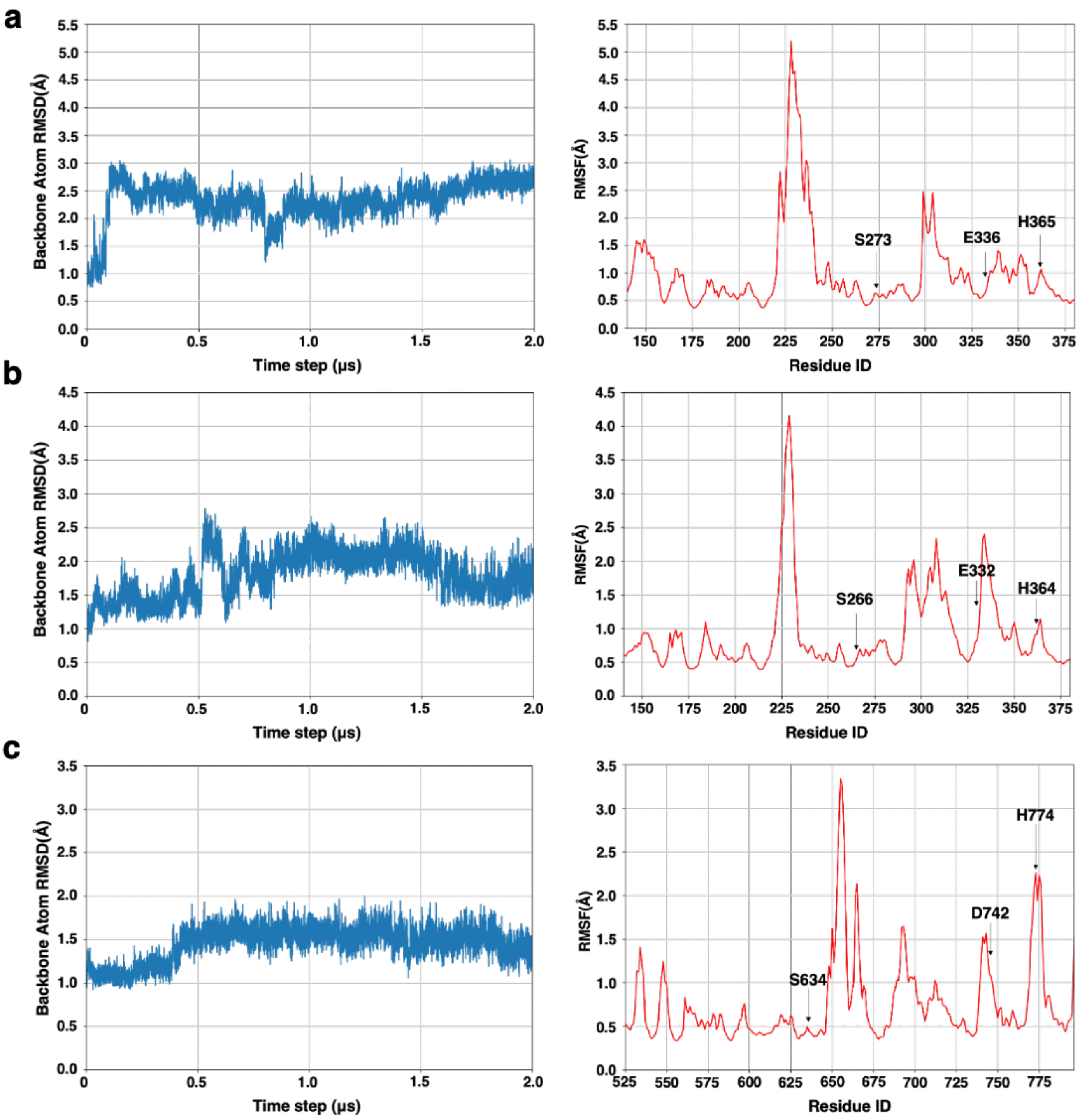
a
Backbone Atom RMSD(Å)
Time step (μs)
RMSF(Å)
Residue ID
S273
E336
H365
b
Backbone Atom RMSD(Å)
Time step (μs)
RMSF(Å)
Residue ID
S266
E332
H364
c
Backbone Atom RMSD(Å)
Time step (μs)
RMSF(Å)
Residue ID
S634
D742
H774

Supplementary Fig 15. RMSD and RMSF analysis of esterase domains of three esterase enzymes (Bi1033-CE1, Bi1039-CE1, and BeGH43/FAE in **a**, **b**, and **c**, respectively). The three figures in the left panel show root mean square deviations of all three proteins. The three figures in the right panel represent root mean square fluctuations of all three proteins. Approximate positions of catalytic triad residues are indicated by an arrow. The analysis was done on 2 μs of simulation data on each protein. For Bi1033-CE1 and Bi1039-CE1, first 15 residues were omitted from calculations because of the large fluctuation of these residues.

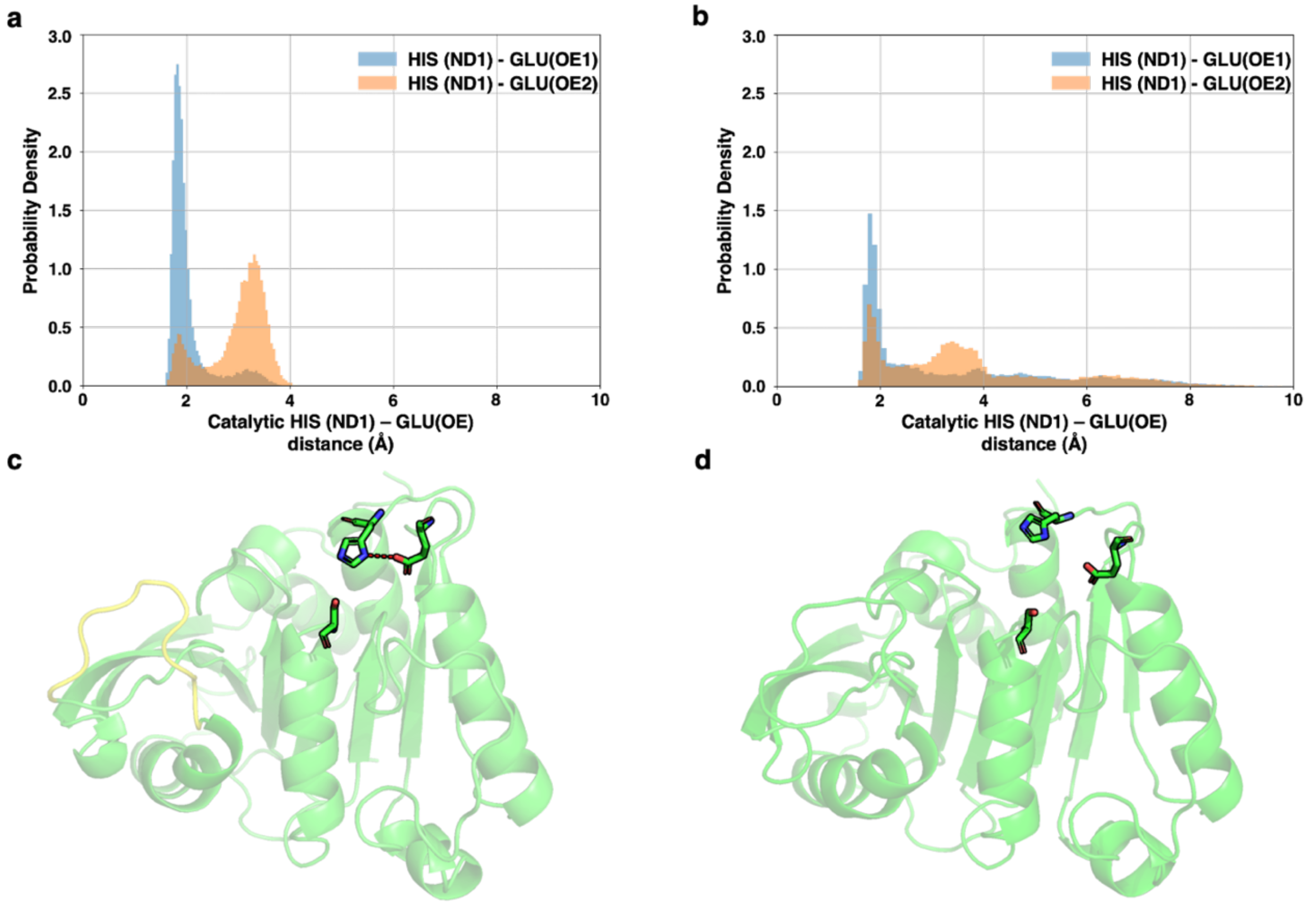

Supplementary Fig. 16. **Higher stability in acid-base hydrogen bonding may lead to higher catalytic activity. a, b** Distance between HIS (ND1) and GLU (OE1, color: Blue; OE2, color: Orange) atoms are represented as a 1-D histogram with increasing mean distance from left (Bi1033-CE1) to right (Bi1039-CE1). **c, d** Cartoon representation of esterase domain of Bi1039-CE1. Catalytic triad residues are shown as stick. Hydrogen Bond between HIS-GLU residue are shown as red dot. In **c**, hydrogen bond between base (HIS) and acid exists but in **d,** base (HIS) moves away from the acid molecule.

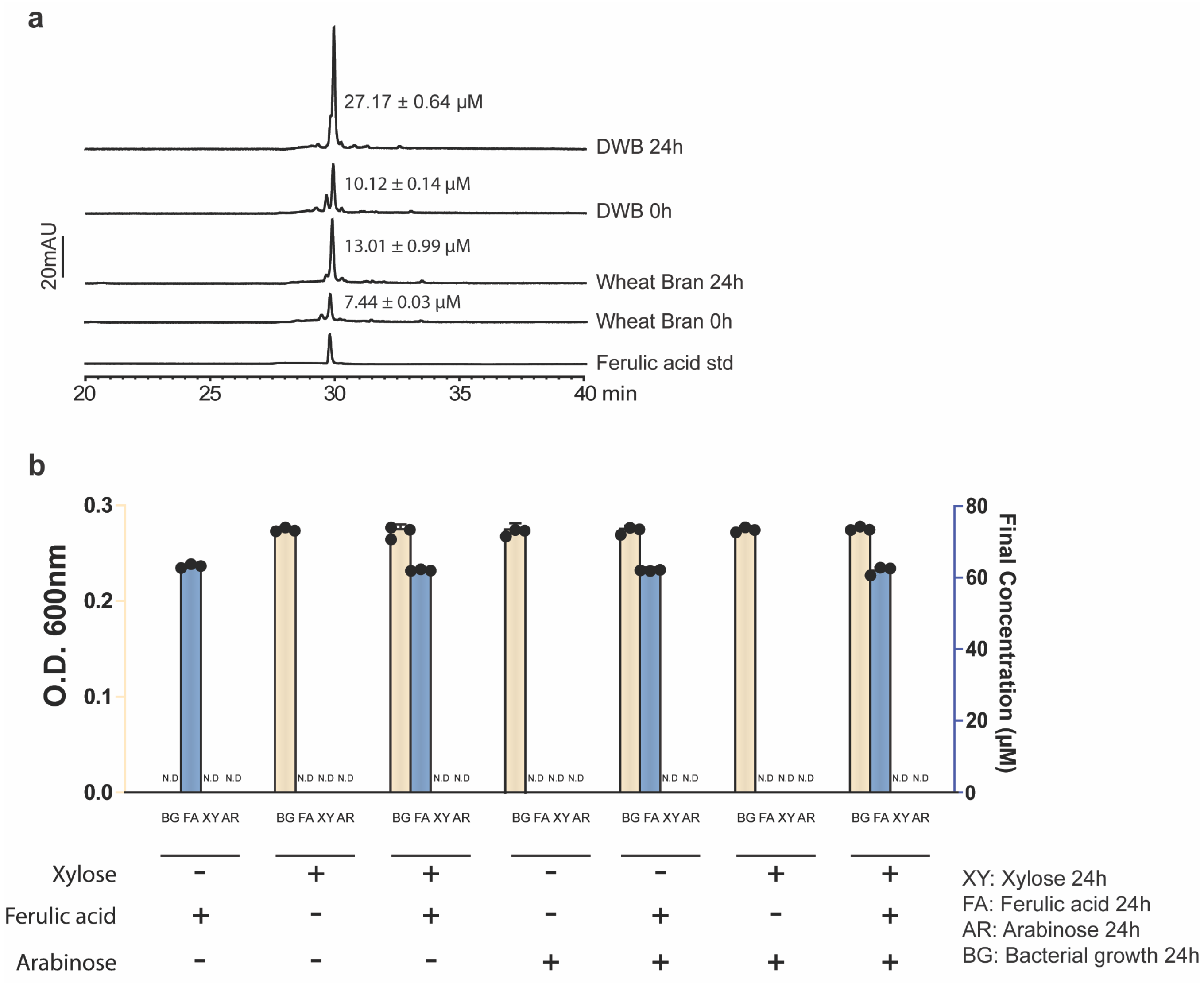

Supplementary Fig. 17. Accumulation of ferulic acid in the spent medium of *B. intestinalis* during growth on arabinoxylan. a HPLC-DAD chromatograms of *B. intestinalis* spent medium during growth on wheat bran or de-starched wheat bran (DWB) as sole carbon source, showing accumulation of ferulic acid in the medium after bacterial culture. The y-axis is absorbance in milli-absorbance units (mAU). **b** Growth of *B. intestinalis* in minimal medium containing ferulic acid as sole carbon source or supplemented with different monosaccharides from arabinoxylan, suggesting the lack of utilization of the phenolic compound by the bacterium. BG: bacterial growth; FA: ferulic acid; XY: xylose; AR: arabinose. In **a**, the results are presented as the mean ± the standard deviation of three independent reactions (n=3), and the traces are representatives of the three different reactions. In **b**, the bars indicate means ± standard deviations of three independent reactions (n=3). ND: not detected. The source data underlying Supplementary Fig. 17**a**-**b** are provided in the Source Data file.

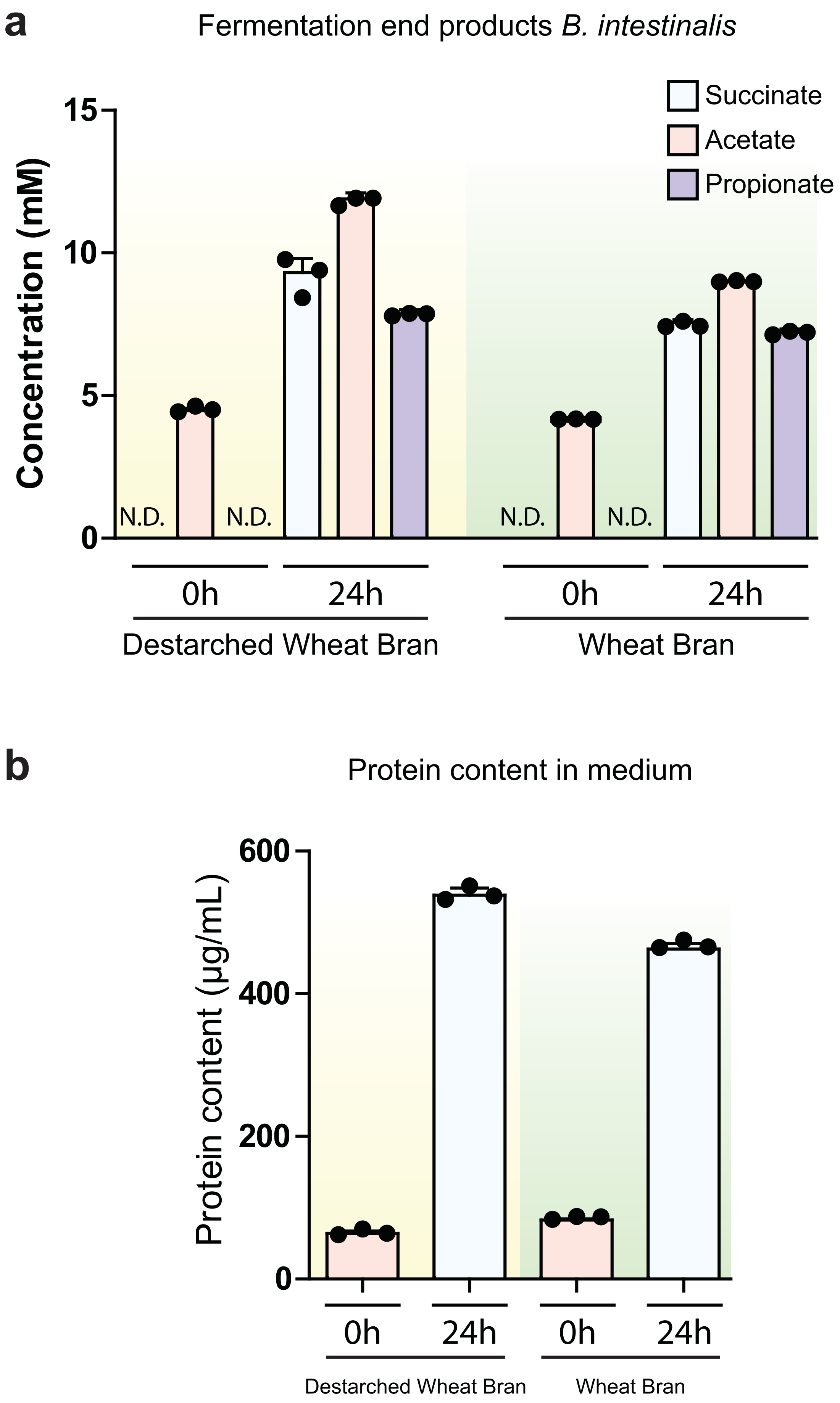
a
Fermentation end products *B. intestinalis*
Succinate
Acetate
Propionate
Concentration (mM)
15
10
5
0
N.D.
N.D.
N.D.
N.D.
0h
24h
0h
24h
Destarched Wheat Bran
Wheat Bran
b
Protein content in medium
Protein content (μg/mL)
600
400
200
0
0h
24h
0h
24h
Destarched Wheat Bran
Wheat Bran

Supplementary Fig. 18. The end products during growth of *B. intestinalis* in a minimal medium containing wheat bran or de-starched wheat bran as a sole carbon source. a The major end products of fermentation of *B. intestinalis* cells grown on either substrate for 24 hours. **b** Protein accumulation in the medium containing either substrate after culturing with *B. intestinalis* cells for 24 hours. In **a** and **b**, the bars indicate means ± standard deviations of three independent reactions (n=3). The source data underlying Supplementary Figs. 18**a**-**b** are provided in the Source Data file.

Supplementary Table 1: X-ray data collection and refinement statistics.

Protein	Bi1033 (dimer/ASU)	Bi1033 (monomer/ASU)	BeGH43-FAE	Bi1039
PDB ID	6MOU	6MOT	6MLY	6NE9
Resolution (Å)	76.37 - 2.24 (2.32 - 2.24)	86.57 - 1.71 (1.77 - 1.71)	48.16 - 2.7 (2.8 - 2.7)	28.6 - 1.74 (1.8 - 1.74)
Space group	P 3_1 2 1	P 6_4 2 2	C 1 2 1	C 2 2 2_1
Unit cell (Å)	a=b=95.2, c=202.6; γ=120	a=b=99.9, c=164.204; γ=120	a=254.7,b=93.3, c=214.0, β=123.38	a=116.7, b=126.6, c=110.3
Total reflections	302305 (30106)	2143630 (125664)	383527 (32441)	145030 (2851)
Unique reflections	52004 (5143)	52998 (5201)	113690 (10279)	76952 (2380)
Multiplicity	5.8 (5.9)	40.4 (24.2)	3.4 (3.2)	1.9 (1.2)
Completeness (%)	100.0 (100.0)	100.0 (100.0)	98.8 (90.1)	91.7 (28.6)
Mean I/sigma(I)	7.5 (1.9)	19.3 (2.3)	8.0 (1.2)	15.3 (2.0)
Wilson B-factor	30.9	24.6	49.0	18.2
R_{merge}	0.18 (0.78)	0.14 (1.02)	0.16 (1.03)	0.06 (0.23)
R_{meas}	0.20	0.15	0.19	0.09
CC1/2	0.99 (0.57)	0.99 (0.89)	0.98 (0.52)	0.99 (0.21)
CC*	0.99 (0.85)	1 (0.97)	0.99 (0.83)	0.99 (0.59)
R_{work}	0.19 (0.30)	0.17 (0.34)	0.22 (0.29)	0.18 (0.28)
R_{free}	0.24 (0.38)	0.19 (0.33)	0.27 (0.36)	0.22 (0.35)
Number non-hydrogen atoms	6163	3080	24144	6165
macromolecules	5702	2758	23692	5568
ligands	16	16	56	110
water	445	306	396	487
Protein residues	713	345	3029	705
RMS(bonds) Å	0.01	0.01	0.01	0.004
RMS(angles) °	1.2	1.2	1.3	0.9
Ramachandran favored (%)	97	97	93	99
Ramachandran outliers (%)	0	0	0.27	0
Clashscore	6.13	2.38	4.32	2.59
<u>Average B-factor</u>	32.7	32	64.8	24.1
macromolecules	32.4	30.6	64.9	23.1
ligands	39.8	40.8	57.9	35.7
solvent	36	44.3	62.2	33

Supplementary Table 2. Comparison of the secondary structure the wild-type and mutant BeGH43/FAE, Bi1033-CE1 and Bi1039-CE1 proteins.

	Helix (%)	Strand (%)	Turn (%)	Unordered (%)
**BeGH43/FAE**				
wt	31.0 ± 0.00	25.5 ± 0.70	19.0 ± 2.82	24.5 ± 2.12
S634A	31.5 ± 0.70	25.0 ± 2.82	18.5 ± 2.12	25.0 ± 4.24
H744A	30.5 ± 0.70	27.0 ± 0.00	21.0 ± 1.41	21.5 ± 2.12
D742A	31.5 ± 2.12	27.0 ± 1.41	20.5 ± 0.70	21.0 ± 2.82
**Bi1033-CE1**				
wt$^{\alpha}$	27.3 ± 1.52	30.0 ± 2.64	15.3 ± 1.52	27.3 ± 5.03
S273A$^{\alpha}$	31.0 ± 2.64	31.3 ± 0.57	14.0 ± 1.00	23.6 ± 3.51
H365A$^{\alpha}$	24.0 ± 2.64	35.6 ± 6.50	16.6 ± 3.51	23.6 ± 6.65
E336A$^{\alpha}$	28.0 ± 3.00	34.0 ± 3.00	12.3 ± 2.51	25.6 ± 3.51
**Bi1039-CE1**				
wt$^{\alpha}$	30.6 ± 1.52	30.6 ± 1.15	11.3 ± 1.15	27.3 ± 0.57
S266A$^{\alpha}$	30.6 ± 2.08	30.0 ± 1.73	10.6 ± 2.08	28.6 ± 1.52
H364A$^{\alpha}$	31.0 ± 1.00	29.6 ± 1.15	12.0 ± 1.00	27.3 ± 0.57
E332A$^{\alpha}$	30.3 ± 3.51	31.6 ± 3.21	10.0 ± 1.00	28.0 ± 0.99

$^{\alpha}$: No statistical significant difference between each mutant and the wild-type (wt). Statistical analyses were not carried out for the BeGH43/FAE data because they come from only two readings. The source data underlying Supplementary Table 2 are provided in the Source Data file.

Supplementary Table 3: Primers used to amplify the genes encoding putative esterase and putative glycosyl hydrolases.

Primer (5'-3')	Gene	Sequence[a]
Bi1033For	BACINT_01033	catatgCAACAGCAAGATTTTCCGGCAGGAAC
Bi1033Rev		ctcgagTTATTTCGTTTTAAATAATAGGGGAGCAAATTCTTTCAGGC
Bi1035For	BACINT_01035	catatgCAAATCGGCACTCCATACATCCACGATC
Bi1035Rev		ctcgagCTAATGGTCGCGGAAATTCCATTTGGAATTG
Bi1038For	BACINT_01038	catatgTTGAATAGAATGAAAAAGCTGTTATTATTTATCGCATGCTTG
Bi1038Rev		ctcgagTTACTTCTTAAATAAATTCGGTAAAAATTCATTCAGACATCTGC
Bi1039For	BACINT_01039	catatgCAGACAGTGGAGGATTTCAAACCATCG
Bi1039Rev		ctcgagTTATTTAAAAAGAAGCGGAGCAAACTCATTCAATGC
Bi1040For	BACINT_01040	catatgCAGATTACGCAATGGACTGATATCAACTATGC
Bi1040Rev		ctcgagTTAATAAAGGGTATAAAACTGTTTTTGAGGAGGATTTTTC
Bi1041For	BACINT_01041	catATGAAGATACTGTTTCATTTCACAATAACTCTGTTCG
Bi1041Rev		ctcgagCTAAGGATGATATTTCCCCAGTATTCTAATTTCGTCTC
Bi1042For	BACINT_01042	catatgCAAACATTGCCGTATCAGAATCCTGAACTAAG
Bi1042Rev		ctcgagTTATTGTAAAGTGACTTTGACAGATTGCAGGTC
Bi1043For	BACINT_01043	catatgCAGAATCCCATTATTACGGATCAGTTCACTG
Bi1043Rev		ctcgagTTATTGAAAACTGATCCAGTCGATTTCAACTTTACC
BeGH43/FaeFor	HMPREF1016_RS0111555	ccgggatccCAAAAGCCTGCAACTAATCCTGTGA
BeGH43/FaeRev		ccgctcgagTCATTTAATGTCATCGCATTTTATCGGCC

Supplementary Table 4. Primers used for Reverse Transcriptase quantitative PCR in *B. intestinalis*, *B. cellulosilyticus*, and *B. oleiciplenus.*

**Primer (5'-3')**	**Gene**	**Sequence**
Bi1036For	BACINT_01036	TGATTCTAACTACACTCTCTTTGGTG
Bi1036Rev		TTGACAGGAGTAACGTTCATGTAAGC
Bi1037For	BACINT_01037	TATCAATCATGAGTTTGCTCACAAAATG
Bi1037Rev		TTCGTCAAATTCTTTATCTGCGCG
Bi16sFor	Bi16s rDNA	GGAGCGTAGGCGGATTATTAAG
Bi16sRev		GGAGCGTAGGCGGATTATTAAG
Bc2149For	BACCELL_02149	GAATGGGCAGGTAACAGAAA
Bc2149Rev		GGATGGCAACCGTACAGATAG
Bc2148For	BACCELL_02148	CACTCCAGACGAGTTCTTATAC
Bc2148Rev		GGCCTTCTGTACTCTTGATACC
Bc16sFor	Bc16s rDNA	AGCAACACAATGCTATG
Bc16sRev		CACGTAAACCACTTTCTT
Bo2534For	HMPREF9447_02534	CGTATCCGGCTTCACCTATTC
Bo2534Rev		GTCGAAACCTACACCCATATCC
Bo2533For	HMPREF9447_02533	GATGCGCTGGCATAACAATAC
Bo2533Rev		CTCTACTCGGAATCGGAAGAATG
Bo16sFor	Bo16s rDNA	CCATTCATTGGGCATAG
Bo16sRev		CGTACTTTCTTACCGATAC

Supplementary Table 5. Primers used for site directed mutagenesis in BeGH43/FAE, Bi1033-CE1, and Bi1039-CE1.

**Primer (5'-3')**	**Gene**	**Sequence[a]**
BeGH43/Fae S634A For	BeGH43/Fae	TACCGGCTTT**gca**TCGGGTGGAC
BeGH43/Fae S634A Rev		ATTCCTATAAAAGAAGTATCCAACTTATACTTGGC
BeGH43/Fae D742A For	BeGH43/Fae	TGGTGAAGCG**gcc**ACTGTGGTTC
BeGH43/Fae D742A Rev		TGAATAACAATAAATTTAGGATCGTTTTTATCTATATAAGTAATG
BeGH43/Fae H774A For	BeGH43/Fae	CGGCGGACAA**gct**GGTCCTGTCAC
BeGH43/Fae H774A Rev		GGAACAGAAATAAATTCTTCCAAAC
Bi1033-CE1 S273A For	Bi1033-CE1	GGCCGGACTT**gcc**TGGGGTGGAC
Bi1033-CE1 S273A Rev		ATGGCGCGATTATCCCTATCGGTCAG
Bi1033-CE1 E336A For	Bi1033-CE1	CGGTTCGGAA**gca**CATCCGGAAA
Bi1033-CE1 E336A Rev		ATGCCCAGGAAGAAGACG
Bi1033-CE1 H365A For	Bi1033-CE1	GGGCACTGCC**gcc**GAGTTCCTCA
Bi1033-CE1 H365A Rev		GGCGATTCGTAATAGATCG
Bi1039-CE1 S266A For	Bi1039-CE1	TGCAGGATTG**gcg**ATGGGGGCTA
Bi1039-CE1 S266A Rev		ATGGCGCGGTCTTCACGAC
Bi1039-CE1 E332A For	Bi1039-CE1	TGGTACGGCC**gca**CCGCATCCTT
Bi1039-CE1 E332A Rev		AGTCCCAGAAAGAAAACCTTAAACTGAACGTTTAC
Bi1039-CE1 H364A For	Bi1039-CE1	CGATACGGCC**gct**GAATGGCTCAC
Bi1039-CE1 H364A Rev		GGAGATTCATAATACACATATTTAATTC

a: Nucleotides mutated are in lowercase and bolded

References:

1 Colquhoun, I. J., Ralet, M. C., Thibault, J. F., Faulds, C. B. & Williamson, G. Structure identification of feruloylated oligosaccharides from sugar-beet pulp by NMR spectroscopy. *Carbohydr. Res.* **263**, 243-256 (1994).
