# Supplementary material for: Degradation of complex arabinoxylans by human colonic Bacteroidetes
Source: Nat Commun. 2021 Jan 19;12:459. doi: 10.1038/s41467-020-20737-5 (PMC7815789; doi:10.1038/s41467-020-20737-5)
Supplement: Supplementary file 3 — Descriptions of Additional Supplementary Files [file 41467_2020_20737_MOESM3_ESM.pdf]

Descriptions of Additional Supplementary Files

Supplementary Data 1

Description: A) Fold change comparison of the top 50 proteins in B. intestinalis during growth on insoluble wheat arabinoxylan (InWAX) compared to a monosaccharide mixture. B) Fold change comparison of the top 50 proteins in B. cellulosilyticus during growth on insoluble wheat arabinoxylan (InWAX) compared to a monosaccharide mixture. C) Fold change comparison of the top 50 proteins in B. oleiciplenus during growth on insoluble wheat arabinoxylan (InWAX) compared to a monosaccharide mixture.

Supplementary Data 2

Description: Fold change comparison of known arabinoxylan targeting PUL of B. intestinalis during growth on soluble (sWAX) or insoluble wheat arabinoxylan (InWAX) compared to a monosaccharide mixture.
