# Supplementary material for: Degradation of complex arabinoxylans by human colonic Bacteroidetes
Source: Nat Commun. 2021 Jan 19;12:459. doi: 10.1038/s41467-020-20737-5 (PMC7815789; doi:10.1038/s41467-020-20737-5)
Supplement: Supplementary file 6 — Reporting Summary [file 41467_2020_20737_MOESM6_ESM.pdf]

nature research

Corresponding author(s): Isaac Cann
Last updated by author(s): Dec 2, 2020

Reporting Summary

Nature Research wishes to improve the reproducibility of the work that we publish. This form provides structure for consistency and transparency in reporting. For further information on Nature Research policies, see our Editorial Policies and the Editorial Policy Checklist.

Statistics

For all statistical analyses, confirm that the following items are present in the figure legend, table legend, main text, or Methods section.

n/a	Confirmed	
☐	☒	The exact sample size (n) for each experimental group/condition, given as a discrete number and unit of measurement
☐	☒	A statement on whether measurements were taken from distinct samples or whether the same sample was measured repeatedly
☐	☒	The statistical test(s) used AND whether they are one- or two-sided<br>*Only common tests should be described solely by name; describe more complex techniques in the Methods section.*
☒	☐	A description of all covariates tested
☒	☐	A description of any assumptions or corrections, such as tests of normality and adjustment for multiple comparisons
☐	☒	A full description of the statistical parameters including central tendency (e.g. means) or other basic estimates (e.g. regression coefficient) AND variation (e.g. standard deviation) or associated estimates of uncertainty (e.g. confidence intervals)
☐	☒	For null hypothesis testing, the test statistic (e.g. F, t, r) with confidence intervals, effect sizes, degrees of freedom and P value noted<br>*Give P values as exact values whenever suitable.*
☒	☐	For Bayesian analysis, information on the choice of priors and Markov chain Monte Carlo settings
☒	☐	For hierarchical and complex designs, identification of the appropriate level for tests and full reporting of outcomes
☒	☐	Estimates of effect sizes (e.g. Cohen's d, Pearson's r), indicating how they were calculated

Our web collection on statistics for biologists contains articles on many of the points above.

Software and code

Policy information about availability of computer code

Data collection	No software was used for data collection

Data analysis

Genome-wide alignment was done with the license based software Geneious Prime. RNAseq analysis were performed with open sourced packages described in the methods section (Trimmomatic v0.33, BowTie2 v2.3.3.1, htseq count (release_0.9.1), edgeR package v3.6, R v3.4.2, and Rstudio v1.1.383).
Signal peptides and lipoproteins were predicted using an online server SignalP v5.0 and Lipo v1.0, respectively.
X-ray data: X-ray data were processed and scaled with Xia2 within the CCP4 (v7.0) suite of programs or HKL2000 (v720-Mac). Structure determination and refinement: AutoSol, AutoBuild, Phenix.refine, within Phenix (1.15-2. 3478); MrBUMP and Phaser within ccp4 (v7.0) Manual model building in Coot (v 0.8.9.1).
Circular Dichroism analyses were carried out with the online server DICHROWEB and reference is provided in the methods section (no version is provided for this program).
Graphpad v8.4.3 was used for statistical analysis.
Molecular Dynamics Simulations were carried out with the following softwares:
Addition of missing amino acids in protein structures: MODELLER v9.21; protein solvation with PACKMOL v18.169; Ferulic acid model was built with MarvinSketch v19.23 and parameterized using AMBER GAFF forcefield; The esterase enzymes were modeled using Amber ff14SB forcefield. Movement of hydrogen atoms were contained by SHAKE algorithm (no versions provided online, but reference provided in the manuscript); Sampling bias in the simulations were removed by constructing a Markov state Model (MSM) using the Python package PyEMMA 2.5.6; Chapman-Kolmogorov test was performed to validate the Makovian behaviour of the MSMs; trajectory data were analyzed using CPPTRAJ v18.01 module in AMBER18 and the Python package MDTraj v1.9.3. The 2-D plots were visualized and analyzed using VDM v1.9.3 and PYMOL v2.1; pocket volume calculations were performed with the Python package POVME v3.0. Some softwares do not have available version numbers, K-means algorithm, Shake algorithm, and Matplotlib. However, the references for each of the data analyses approaches are listed in the methods section.

For manuscripts utilizing custom algorithms or software that are central to the research but not yet described in published literature, software must be made available to editors and reviewers. We strongly encourage code deposition in a community repository (e.g. GitHub). See the Nature Research guidelines for submitting code & software for further information.

Data

Policy information about availability of data

All manuscripts must include a data availability statement. This statement should provide the following information, where applicable:

- Accession codes, unique identifiers, or web links for publicly available datasets
- A list of figures that have associated raw data
- A description of any restrictions on data availability

We have provided all the accession numbers including RNAseq data and structure PDB identifiers in the Data availability statement.
X-ray structure data including coordinates and structure factors have been deposited in the Protein Data Bank (PDB) and are readily accessible to the public under accession codes 6MOU, 6MLY, 6NEP, and 6MOT. Below, we provide more information and readily accessible links.

The RNASeq data are deposited at the NCBI GEO under the accession numbers GSE161471 (raw reads and differential expression files). The Sequence Read Archives (SRA) containing the raw reads of the individual samples is at [https://www.ncbi.nlm.nih.gov/Traces/study/?acc=PRJNA678359&o=acc_s%3Aa].

The Protein Databank (PDB) IDs of the structures presented in this study are as follows: 6MOU (BACINT_01033-CE1 dimer) [https://www.rcsb.org/structure/6MOU], 6MOT(BACINT_01033-CE1 monomer) [https://www.rcsb.org/structure/6MOT], 6NE9(BACINT_01039-CE1) [https://www.rcsb.org/structure/6NE9], and 6MLY (BEGH43/FAE) [https://www.rcsb.org/structure/6MLY]

Other PDB identifiers used in this work.
Humicola insolens GH43 glycoside hydrolase PDB 3ZXL (https://www.rcsb.org/structure/3ZXL)
Geobacillus thermoleovorans beta-1,4-xylosidase PBD 5Z5I (https://www.rcsb.org/structure/5Z5I)
Lactobacillus plantarum tannase PBD 4JUI (https://www.rcsb.org/structure/4JUI)
Clostridium thermocellum xylanase 1JT2 (https://www.rcsb.org/structure/1JT2)

Databases used in the reported research.
The Polysaccharide-Utilization Loci DataBase (PULDB) server (http://www.cazy.org/PULDB/)
Genbank protein database (https://www.ncbi.nlm.nih.gov/protein)

All datasets and recombinant plasmids generated during and/or analyzed during the current study are also available from the corresponding author (Isaac Cann) on reasonable request. Source data are provided with this paper.

Field-specific reporting

Please select the one below that is the best fit for your research. If you are not sure, read the appropriate sections before making your selection.

- [x] Life sciences
- [ ] Behavioural & social sciences
- [ ] Ecological, evolutionary & environmental sciences

For a reference copy of the document with all sections, see nature.com/documents/nr-reporting-summary-flat.pdf

Life sciences study design

All studies must disclose on these points even when the disclosure is negative.

Sample size	Sample size (replicates) were based on previous experience on our published enzymatic work and also on our published RNAseq experiments, including the following:<br>D. Dodd, S. A. Kocherginskaya, M. A. Spies, K. E. Beery, C.A. Abbas, R.I Mackie, and I.K.O. Cann. 2009. Biochemical analysis of a β-D-xylosidase and a bifunctional xylanase-ferulic acid esterase from a xylanolytic gene cluster in Prevotella ruminicola 23. J. Bacteriol. 191: 3328-3338. PMID 19304844.<br><br>D. Dodd, Y. H. Moon, K. Swaminathan, R.I. Mackie, and I.K.O. Cann. 2010. Transcriptomic analyses of xylan degradation by Prevotella bryantii and insights into energy acquisition by xylanaolytic bacteroidetes. J. Biol. Chem. 285:30261-30273. PMID 20622018.<br><br>M. Zhang, J.R. Chekan, D. Dodd, P.Y. Hong, L. Radlinski, V. Revindran, S.K. Nair, R.I. Mackie, and I. Cann. 2014. Xylan utilization in human gut commensal bacteria is orchestrated by unique modular organization of polysaccharide-degrading enzymes. Proc. Natl Acad. Sci. U S A. 111:E3708-3717. PMID 25136124.
Data exclusions	There was no exclusion data
Replication	All biochemical reactions, such as enzyme assays, and also RNAseq involved two or more independent reactions and the number of reactions have been clearly stated in the legends for the figures, and the data were presented as means ± standard deviations, where applicable. Statistics were not carried out on any experiments where there were only duplicate independent reactions.
Randomization	The experiments, which are mostly based on transcriptional analysis, protein purification, enzymology, x-ray crystallography and molecular dynamics simulations, did not require randomization.
Blinding	Blinding was not required or done in our experiments.

Reporting for specific materials, systems and methods

We require information from authors about some types of materials, experimental systems and methods used in many studies. Here, indicate whether each material, system or method listed is relevant to your study. If you are not sure if a list item applies to your research, read the appropriate section before selecting a response.

Materials & experimental systems

n/a	Involved in the study
☒	☐ Antibodies
☒	☐ Eukaryotic cell lines
☒	☐ Palaeontology and archaeology
☒	☐ Animals and other organisms
☒	☐ Human research participants
☒	☐ Clinical data
☒	☐ Dual use research of concern

Methods

n/a	Involved in the study
☒	☐ ChIP-seq
☒	☐ Flow cytometry
☒	☐ MRI-based neuroimaging
